# DeepComBat: A statistically motivated, hyperparameter‐robust, deep learning approach to harmonization of neuroimaging data

**DOI:** 10.1002/hbm.26708

**Published:** 2024-07-26

**Authors:** Fengling Hu, Alfredo Lucas, Andrew A. Chen, Kyle Coleman, Hannah Horng, Raymond W. S. Ng, Nicholas J. Tustison, Kathryn A. Davis, Haochang Shou, Mingyao Li, Russell T. Shinohara, The Alzheimer's Disease Neuroimaging Initiative

**Affiliations:** ^1^ Penn Statistics in Imaging and Visualization Endeavor (PennSIVE), Department of Biostatistics, Epidemiology, and Informatics Perelman School of Medicine, University of Pennsylvania Philadelphia Pennsylvania USA; ^2^ Center for Neuroengineering and Therapeutics, Department of Engineering University of Pennsylvania Philadelphia Pennsylvania USA; ^3^ Statistical Center for Single‐Cell and Spatial Genomics Perelman School of Medicine, University of Pennsylvania Philadelphia Pennsylvania USA; ^4^ Perelman School of Medicine, University of Pennsylvania Philadelphia Pennsylvania USA; ^5^ Department of Radiology and Medical Imaging University of Virginia Charlottesville Virginia USA; ^6^ Department of Neurology Perelman School of Medicine, University of Pennsylvania Philadelphia Pennsylvania USA; ^7^ Center for Biomedical Image Computing and Analytics (CBICA) Perelman School of Medicine Philadelphia Pennsylvania USA

**Keywords:** deep learning, image harmonization, neuroimaging, reproducibility

## Abstract

Neuroimaging data acquired using multiple scanners or protocols are increasingly available. However, such data exhibit technical artifacts across batches which introduce confounding and decrease reproducibility. This is especially true when multi‐batch data are analyzed using complex downstream models which are more likely to pick up on and implicitly incorporate batch‐related information. Previously proposed image harmonization methods have sought to remove these batch effects; however, batch effects remain detectable in the data after applying these methods. We present DeepComBat, a deep learning harmonization method based on a conditional variational autoencoder and the ComBat method. DeepComBat combines the strengths of statistical and deep learning methods in order to account for the multivariate relationships between features while simultaneously relaxing strong assumptions made by previous deep learning harmonization methods. As a result, DeepComBat can perform multivariate harmonization while preserving data structure and avoiding the introduction of synthetic artifacts. We apply this method to cortical thickness measurements from a cognitive‐aging cohort and show DeepComBat qualitatively and quantitatively outperforms existing methods in removing batch effects while preserving biological heterogeneity. Additionally, DeepComBat provides a new perspective for statistically motivated deep learning harmonization methods.

## Graphical abstract

Neuroimaging data from different manufacturers exhibit technical artifacts, introducing confounding and decreasing reproducibility. We present DeepComBat, a deep learning harmonization method using a conditional variational autoencoder and the ComBat method. DeepComBat combines strengths of statistical and deep learning methods to correct batch effects while relaxing assumptions made by previous methods.

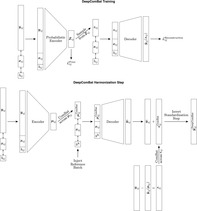

**Practitioner Points**

- Batch effects are present in datasets collected across multiple scanners or sites, and presence of these batch effects may decrease reproducibility and generalizability.
- DeepComBat offers improved removal of nonlinear batch effects in multivariate settings.
- DeepComBat relies on fewer strong assumptions compared to current methods and offers a new perspective on deep learning‐based harmonization methods.

## 1 INTRODUCTION

There is increasing need for larger sample sizes in human magnetic resonance imaging (MRI) studies to detect small effect sizes, train accurate prediction models, improve generalizability, and more. This has led to more interest in multi‐batch studies, where subjects are imaged across multiple settings—different research sites, scanner manufacturers, acquisition settings, magnet strengths, and more—and then aggregated together (Bethlehem et al., 2022; Casey et al., 2018; Di Martino et al., 2014; Marek et al., 2022; Mueller et al., 2005; Trivedi et al., 2016; Van Essen et al., 2013). Multi‐batch studies overcome limitations of single site studies, which are often unable to recruit sufficiently large or representative samples to achieve study goals; however, multi‐batch study designs introduce non‐biological, technical variability between subjects imaged from different batches due to differences in acquisition, scanner manufacturer, magnet strength, post‐processing, and more (Badhwar et al., 2020; Han et al., 2006; Jovicich et al., 2006; Takao et al., 2011; Takao et al., 2014). Such technical variability is often referred to as “scanner effects” or “batch effects” and, if not appropriately addressed, may result in invalid, non‐reproducible, or non‐generalizable study results. Post‐acquisition removal of these batch effects, known as image harmonization, is a promising approach for mitigating these issues (Hu et al., 2023).

Harmonization of image‐derived features, such as cortical thicknesses, functional connectivity values, radiomics features, and more has been extensively studied. Fortin et al. (2017) showed that the ComBat model, adapted from the genomics setting, could effectively remove batch effects by modeling them univariately as additive differences in means and as multiplicative differences in variances of residuals (Johnson et al., 2007). This model has also been extended to unique data settings, such as those where covariate effects are nonlinear, longitudinal data is present, decentralized learning is required, multiple batch variables should be corrected for, or traveling subjects are available (Bayer et al., 2022; Bostami et al., 2022; Chen, Luo, et al., 2022; Horng et al., 2022; Maikusa et al., 2021; Pomponio et al., 2020). In applied studies, ComBat‐family methods have been widely used and shown to improve inference and generalizability of results (Acquitter et al., 2022; Bartlett et al., 2018; Bourbonne et al., 2021; Crombé et al., 2020; Fortin et al., 2018; Marek et al., 2019; Yu et al., 2018). This may be especially true in mass univariate inference settings, where biological effects are modeled at the individual feature level, since this setting matches the data assumptions made by the ComBat model.

However, in studies where feature‐level data are used in a highly multivariate manner, univariate harmonization approaches may be insufficient. For example, as imaging researchers have become more interested in complex prediction efforts, multivariate feature datasets are used as inputs to predict an outcome of interest. In these settings, state‐of‐the‐art machine learning (ML) algorithms are often used as powerful approaches that are able to jointly leverage the multivariate distribution of features, accounting for complex nonlinear and interaction effects (Hu et al., 2023; Koutsouleris et al., 2014; Smith et al., 2017; Wager et al., 2013). Batch effects that exist in the interactions between features may also be picked up by these ML algorithms, which can lead to decreased generalizability of these models and overfitting of model parameters on batch effects, especially when batch status is a relevant confounder for the outcome. Thus, recent efforts in feature‐level harmonization have attempted to detect and mitigate such multivariate batch effects.

From the statistical perspective, recently proposed methods for multivariate harmonization have included CovBat (Chen et al., 2022), Bayesian factor regression (BFR, Avalos‐Pacheco et al., 2022), and UNIFAC (Zhang et al., 2022). Like ComBat, these models assume batch effects can be effectively modeled through the combination of low‐rank additive and multiplicative effects. However, instead of modeling batch effects solely in a univariate manner, CovBat additionally assumes batch effects to be present in the covariance structure of model residuals, while BFR and UNIFAC assume additive batch effects to be present in the direction of multivariate latent factors. Additionally, while ComBat, CovBat, and UNIFAC all seek to ultimately produce a dataset of harmonized features, BFR instead learns a low‐dimensional representation of the original features where batch effects have been removed; BFR does not map this low‐dimensional representation back to the feature space.

From the deep learning perspective, feature‐level multivariate harmonization methods have leveraged the conditional variational autoencoder (CVAE) architecture, an adaptation of the standard variational autoencoder that attempts to disentangle the latent space distribution from covariates of interest (Kingma & Welling, 2014; Sohn et al., 2015). These models include diffusion CVAE (dcVAE, Moyer et al., 2020) and goal‐specific CVAE (gcVAE, An et al., 2022). In dcVAE, an encoder embeds vector representations of diffusion MRI data as latent space distributions, and the encoder is penalized when batch‐specific information is present in the latent space representation. Then, the decoder is given these latent space distributions along with explicit batch information and trained to reconstruct the original input. Through this process, dcVAE assumes that the encoder can learn to remove batch effects and the decoder can accurately reconstruct the original data while removing batch effects. However, An et al. (2022) noted that dcVAE removes biological information of interest. They proposed gcVAE could recover this biological information by fine‐tuning the dcVAE decoder such that the decoder could not only accurately reconstruct the input but could also retain biological information of interest in the reconstruction. gcVAE encourages this behavior by adding a pre‐trained neural network classifier to the end of dcVAE that uses decoder output to predict biological covariates of interest. Classifier success is rewarded in the loss function. Additionally, a deep learning network called flow‐based structural causal model has been explored for feature‐level harmonization (Wang et al., 2021). This method seeks to learn the causal effect of batch, conditional on biological covariates, and sample from the posterior distribution under the counterfactual batch with the goal to improve predictive performance of a model trained in a reference dataset.

Notably, deep learning harmonization methods designed for feature‐level data make a number of strong implicit assumptions. First, deep learning harmonization methods directly use model outputs from the harmonization step as the resulting harmonized data—unmodeled residual terms are unaccounted for, as well as any batch or biological effects in these residuals. Implicitly, this makes the strong assumption that the deep learning method achieves perfect or nearly perfect model fit—that is, the reconstruction loss is zero or nearly zero. This is in contrast to statistical harmonization methods, which tend to estimate batch effects within unmodeled residual as a difference in scale; the residuals are rescaled and added back to the model‐based biological effects to produce the resulting harmonized data. Secondly, deep learning harmonization methods assume that batch and biological effects can be completely disentangled through loss function optimization and choice of network architecture. While this may be easily achievable in isolation, complete disentanglement may be challenging to achieve in conjunction with the implicit nearly perfect model fit assumption. Third, these deep learning methods use loss functions that are limited to harmonization between only two batches—settings where multiple batches exist are infeasible. Finally, deep learning harmonization methods often do not explicitly consider that biological covariates may be imbalanced across batches—in such cases, some differences across batches may actually be due to true biological differences and therefore should not be removed.

We propose a novel deep learning harmonization method, called DeepComBat, that effectively removes multivariate batch effects between two or more batches in a statistically informed manner. Compared to statistical methods such as ComBat and CovBat, DeepComBat removes complex, nonlinear, and multivariate batch effects from the raw data in a way that mitigates detection of batch effects using highly multivariate methods. Compared to other deep learning methods, DeepComBat avoids making the assumptions described above—unmodeled residual terms are reintroduced, a completely disentangled latent space is not required, multiple batches can be harmonized, and model‐based batch effects are removed conditional on biological covariates that may be confounders. To the best of our knowledge, DeepComBat is the first deep learning harmonization method that avoids such assumptions. Additionally, DeepComBat hyperparameters can be easily tuned manually, and DeepComBat can be thought to have a form of “double‐robustness” such that even with poor model fit, reasonable harmonization can still be achieved.

We apply DeepComBat to cortical thickness measurements acquired through the Alzheimer's Disease Neuroimaging Initiative (ADNI) and compare our results to those of other feature‐level harmonization methods where open‐source code was available, namely: ComBat, CovBat, dcVAE (modified for non‐diffusion setting), and gcVAE. Compared to other methods, DeepComBat‐harmonized data retain biological information of interest while containing less batch information. Our results demonstrate the advantage of incorporating statistical ideas into deep learning methods to perform multivariate harmonization.

## 2 METHODS

### 2.1 ADNI dataset and preprocessing

We included 663 unique subjects (381 males) from the ADNI (http://adni.loni.usc.edu/). For each subject, the most recent T1‐weighted (T1w) imaging acquired during the ADNI‐1 phase was used; all included images were acquired between July 2006 and August 2010. Informed consent was obtained for all subjects in the ADNI study. Institutional review boards approved the study at all of the contributing institutions.

We define three batches based on which manufacturer each subject's scanner was from. This included Siemens Healthineers (*n* = 280), General Electric (GE) Healthcare scanners (*n* = 287), and Philips Medical Systems (*n* = 96). For the purpose of Supplemental Materials where we compare DeepComBat to dcVAE and gcVAE, which can only harmonize between two batches, we define Siemens and non‐Siemens as the two batches. Additionally, we define age, sex, and Alzheimer disease (AD) status (cognitively normal, late mild cognitive impairment [MCI], AD) as biological covariates of interest that may confound the relationship between batch status and T1w imaging—these covariates are known to affect brain structure and also may be associated with scanner manufacturer through differing population demographics across sites. Subject demographics at time of most recent acquisition are presented in Table 1, stratified by these two batches. Notably, there are marked differences in the distribution of sex across the three batches, suggesting that confounding of batch status by subject demographics is plausible, and estimation of batch effects should be conditioned on subject demographics.

**TABLE 1:** Patient demographics at time of acquisition, stratified by batch.

	GE, *N* = 287	Philips, *N* = 96	Siemens, *N* = 280
Age	77.3 (7.1)	76.2 (6.2)	77.8 (6.6)
*Sex*			
Male	171 (60%)	63 (66%)	147 (52%)
Female	116 (40%)	33 (34%)	133 (48%)
*Diagnosis*			
Cognitively normal	82 (29%)	33 (34%)	82 (29%)
Late mild cognitive impairment	144 (50%)	41 (43%)	139 (50%)
Alzheimer disease	61 (21%)	22 (23%)	59 (21%)
Mini‐Mental State Examination Score	25.3 (4.7)	25.0 (5.5)	24.8 (5.5)
Mean (SD); *n* (%)			

Processing of these data was carried out using the Advanced Normalization Tools longitudinal single‐subject template pipeline (Tustison et al., 2019). Briefly, we first downloaded raw T1w images from the ADNI‐1 database, which were acquired using MPRAGE for Siemens and Philips scanners and using a works‐in‐progress version of MPRAGE for GE scanners (Jack Jr. et al., 2010). For each subject, we estimated a single‐subject template using all image timepoints and applied rigid spatial normalization to this template for each timepoint image. Then, each normalized timepoint image is processed using the single‐image cortical thickness pipeline consisting of (1) brain extraction (Avants et al., 2010), (2) denoising (Manjón et al., 2010), (3) N4 bias correction (Tustison et al., 2010), (4) Atropos n‐tissue segmentation (Avants et al., 2011), and (5) registration‐based cortical thickness estimation (Das et al., 2009). Finally, for our analyses, we used cortical thickness values for the 62 Desikan‐Killiany‐Tourville atlas regions such that the feature matrix we sought to harmonize was of dimension 663×62 (Klein & Tourville, 2012). Scan metadata were determined based on information contained within the Digital Imaging and Communications in Medicine headers for each scan.

### 2.2 ComBat model

We first review the ComBat (Combatting Batch Effects) model, which models additive and multiplicative batch effects in an empirical Bayes framework (Fortin et al., 2017; Johnson et al., 2007). This model is used as a building block for DeepComBat. For each subject, let $y_{\mathrm{ij}}={\left[y_{\mathrm{ij}1},\ldots ,y_{\mathrm{ijk}},\ldots ,y_{\mathrm{ijp}}\right]}^{⊺}$ represent the p×1 vector of feature‐level information for that subject, where each $y_{\mathrm{ijk}}$ is a scalar. In this notation, i=1,2,…,B indexes batch; $j=1,2,\ldots ,n_{i}$ indexes subjects within batch i, where $n_{i}$ is the number of subjects acquired in batch i; and k=1,2,…,p indexes features, where p is the total number of features. First, ComBat is fit on each feature individually using the following model:

$$y_{\mathrm{ijk}}=\alpha_{k}+x_{\mathrm{ij}}^{T}\beta_{k}+\gamma_{\mathrm{ik}}+\delta_{\mathrm{ik}}e_{\mathrm{ijk}}$$

where $\alpha_{k}$ is the vector of shared intercepts across batches; $x_{\mathrm{ij}}$ is the vector of subject‐specific biological covariates; $\beta_{k}$ is the vector of regression coefficients for the covariates; $\gamma_{\mathrm{ik}}$ is the vector of mean batch effects for batch i conditional on the covariates; and $\delta_{\mathrm{ik}}$ is the vector of multiplicative batch effects on the residuals. ComBat assumes the errors, $e_{\mathrm{ijk}}$, are distributed $N\left(0,\sigma_{k}^{2}\right)$.

For each individual feature, least‐squares estimates ${\hat{\alpha }}_{k}$ and ${\hat{\beta }}_{k}$ are obtained. Then, to estimate batch effects using empirical Bayes, ComBat assumes the additive batch effects, $\gamma_{\mathrm{ik}}$, are drawn from a normal distribution prior and the multiplicative batch effects, $\delta_{\mathrm{ik}}$, are drawn from an inverse gamma distribution prior. Hyperparameters for these priors are estimated via method of moments using data across all features. Next, for each feature‐level, empirical Bayes estimates, $\gamma_{\mathrm{iv}}^{*}$ and $\delta_{\mathrm{iv}}^{*}$, are obtained as the means of their corresponding posterior distributions. This results in shrinkage estimators for both the additive and multiplicative batch effects such that these effects can be well‐estimated even when within‐batch sample size is small. Finally, estimated batch effects are removed using the following equation:

$$y_{\mathrm{ijk}}^{\text{ComBat}}={\hat{\alpha }}_{k}+x_{\mathrm{ij}}^{T}{\hat{\beta }}_{k}+\frac{1}{\delta_{\mathrm{ik}}^{*}}\left(y_{\mathrm{ijk}}-{\hat{y}}_{\mathrm{ijk}}\right)$$

where ${\hat{y}}_{\mathrm{ijk}}={\hat{\alpha }}_{k}+x_{\mathrm{ij}}^{T}{\hat{\beta }}_{k}+\gamma_{\mathrm{ik}}^{*}$ is the subject‐specific mean as estimated by the ComBat model.

### 2.3 DeepComBat method

We propose two versions of DeepComBat—one for internal harmonization and one external harmonization. We define internal harmonization as harmonization in settings where the entire dataset is available and the goal is to remove confounding batch effects for univariate or multivariate inference. Meanwhile, external harmonization is useful in settings where a subset of the dataset is available for training harmonization methods and prediction models, and future out‐of‐sample data needs to be brought into the dataset. Internal and external DeepComBat are nearly identical, with the exception of how CVAE parameters are optimized, described below.

For internal harmonization, all 663 subjects were included for ComBat, CovBat, and DeepComBat training and harmonization. For external harmonization, the DeepComBat‐harmonized dataset was produced using 10‐fold cross‐validation; in each fold, 90% of the data was used to train DeepComBat parameters, and these parameters were then applied to the remaining 10% of out‐of‐sample subjects. The final out‐of‐sample harmonized dataset was produced by joining all out‐of‐sample harmonized subjects across the 10 folds. The same procedure is used for external harmonization using ComBat and CovBat.

#### 2.3.1 Normalization

Normalization and standardization steps are used to encourage faster convergence of the DeepComBat CVAE. As in the above notation, let $y_{\mathrm{ij}}={\left[y_{\mathrm{ij}1},\ldots ,y_{\mathrm{ijk}},\ldots ,y_{\mathrm{ijp}}\right]}^{⊺}$ represent subject *ij*'s cortical thickness vector, where k indexes features, and $x_{\mathrm{ij}}$ represent the vector of subject‐specific biological covariates. Additionally, let $b_{\mathrm{ij}}$ represent that subject's batch covariate.

In the normalization step, all biological covariates are linearly shifted and scaled across all ij subjects such that they range between 0 and 1. Batch covariates are indicators and are thus already in this range. Additionally, each feature is standardized across all ij subjects such that the overall mean for that feature is 0 and the variance is 1. CVAE training and harmonization steps use this normalized data; however, the linear transformations of features are stored such that they can be inverted, and the harmonized output will remain in the original feature space.

#### 2.3.2 Architecture

**FIGURE 1:**
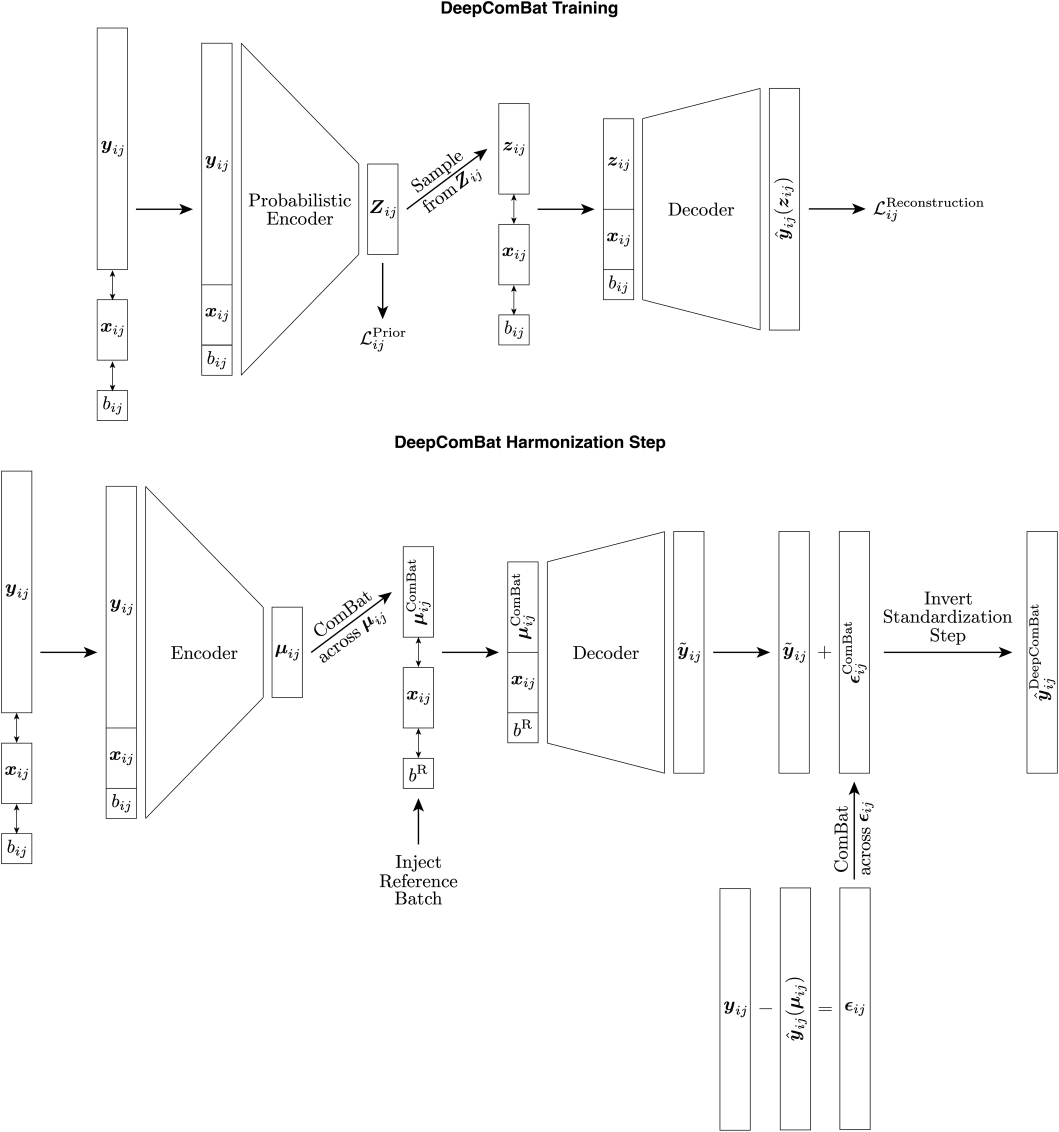
Top: DeepComBat conditional variational autoencoder (CVAE) architecture and loss functions used during training. Bottom: DeepComBat CVAE algorithm used during the harmonization step. At this step, encoder and decoder parameters have been learned during the training step and are frozen. Notation corresponds to that in the main text.

In the CVAE training step, normalized ADNI cortical thickness data are passed through a standard, fully connected CVAE‐style model with the architecture shown in Figure 1. For architectural hyperparameters, the latent space was empirically chosen to be approximately one‐fourth the size of the input vector, rounded to the nearest power of 2—in practice, latent spaces approximately one‐eight or one‐half the size of the input vector also performed similarly. Four hidden layers were used on either side of the latent space to allow for sufficient complexity of the encoder to learn meaningful latent space representations with minimal batch effects and of the decoder to incorporate batch effects in reconstruction. Hidden layer sizes were defined such that each size was approximately halfway between the size of the layers before and after. Hyperbolic tangent (TanH) activation functions were used between layers to allow for nonlinearity—the TanH activation function was empirically found to perform better than rectified linear units in this application.

One iteration through the CVAE for one subject is as follows. First, let the encoder input be defined as the column‐wise concatenation of the column vectors $y_{\mathrm{ij}}$, $x_{\mathrm{ij}}$, and $b_{\mathrm{ij}}$. This encoder input is passed through successive hidden layers until it is eventually encoded into two 16×1 vectors—$\mu_{\mathrm{ij}}=p_{\theta_{1}}\left(y_{\mathrm{ij}},x_{\mathrm{ij}},b_{\mathrm{ij}}\right)$ and $\sigma_{\mathrm{ij}}=p_{\theta_{2}}\left(y_{\mathrm{ij}},x_{\mathrm{ij}},b_{\mathrm{ij}}\right)$, where $p_{\theta_{1}}\left(\cdot ,\cdot ,\cdot \right)$ and $p_{\theta_{2}}\left(\cdot ,\cdot ,\cdot \right)$ represent the encoder functions with neural network parameters $\theta_{1}$ and $\theta_{2}$, respectively. These vectors together define a multivariate normal random variable, $Z_{\mathrm{ij}}∼N\left(\mu_{\mathrm{ij}},\mathrm{diag}\left(\sigma_{\mathrm{ij}}\right)\right)$. This random variable is the output of the encoder and can be thought of as subject *ij*'s latent space representation. Next, to begin the decoding step, a sample is drawn from this random variable using the reparameterization trick in order to obtain $z_{\mathrm{ij}}$ (Kingma & Welling, 2014). As with the encoder input, this sample is column‐wise concatenated with $x_{\mathrm{ij}}$ and $b_{\mathrm{ij}}$ to produce the decoder input. Then, it is passed through the decoder hidden and output layers to obtain a reconstructed feature vector, ${\hat{y}}_{\mathrm{ij}}\left(z_{\mathrm{ij}}\right)=q_{\phi }\left(z_{\mathrm{ij}},x_{\mathrm{ij}},b_{\mathrm{ij}}\right)$, where $q_{\phi }\left(\cdot ,\cdot ,\cdot \right)$ is the decoder function with neural network parameters ϕ. Note that this reconstructed feature vector is a function of the sample from the random variable $Z_{\mathrm{ij}}$ and thus changes each time subject ij is passed through the CVAE. Notably, while it may be unnecessary to provide the encoder with biological and batch covariates, we thought providing such covariates could be useful to the encoder for learning a covariate‐invariant latent space.

Thus, the latent space distribution, $Z_{\mathrm{ij}}$, is a function of the features, $y_{\mathrm{ij}}$, as well as the covariates $x_{\mathrm{ij}}$ and $b_{\mathrm{ij}}$. Similarly, the reconstructed feature vector, ${\hat{y}}_{\mathrm{ij}}\left(z_{\mathrm{ij}}\right)$ is a function of the latent distribution through $z_{\mathrm{ij}}$, as well as the covariates $x_{\mathrm{ij}}$ and $b_{\mathrm{ij}}$. Additionally, by giving the decoder random samples from the latent space distribution, the decoder learns that probabilistically nearby points in the latent space should be mapped to similar outputs in the feature space. That is, the decoder learns to reconstruct the features such that ${\hat{y}}_{\mathrm{ij}}\left(z_{\mathrm{ij}}\right)\approx {\hat{y}}_{\mathrm{ij}}\left(\mu_{\mathrm{ij}}\right)$. The risk of overfitting by the decoder is also minimized, as this random sampling functions as a form of data augmentation with respect to the decoder.

#### 2.3.3 Loss function

The loss function was defined to be the standard CVAE loss function which consists of an autoencoder reconstruction loss component and a Kullback–Leibler (KL) divergence loss component (Kingma & Welling, 2014; Sohn et al., 2015). In the DeepComBat CVAE, this loss function is implemented for each subject as follows:

$$L_{\mathrm{ij}}=L_{\mathrm{ij}}^{\text{Reconstruction}}+\lambda L_{\mathrm{ij}}^{\text{Prior}}=\sum_{k=1}^{p}{\left(y_{\mathrm{ijk}}-{\hat{y}}_{\mathrm{ijk}}\right)}^{2}+\lambda D_{\mathrm{KL}}\left(f\left(Z_{\mathrm{ij}}\right)∥g\left(Z\right)\right)$$

where $L_{\mathrm{ij}}^{\text{Reconstruction}}=\sum_{k=1}^{p}{\left(y_{\mathrm{ijk}}-{\hat{y}}_{\mathrm{ijk}}\right)}^{2}$ is the reconstruction component, $L_{\mathrm{ij}}^{\text{Prior}}=D_{\mathrm{KL}}\left(f\left(Z_{\mathrm{ij}}\right)∥g\left(Z\right)\right)$ is the KL divergence component, and λ is a hyperparameter to weight the relative importance of the two components. The KL divergence component measures the difference between $f\left(Z_{\mathrm{ij}}\right)$, which is the probability density function of the multivariate normal latent space distribution for subject ij, $N\left(\mu_{\mathrm{ij}},\mathrm{diag}\left(\sigma_{\mathrm{ij}}\right)\right)$, and $g\left(Z\right)$, which is defined in DeepComBat to be the probability density function of the standard multivariate normal distribution, $N\left(0,I\right)$. The overall loss function is defined as the sum over all subjects: $L^{\text{Overall}}=\sum_{i=1}^{B}\sum_{j=1}^{n_{i}}L_{\mathrm{ij}}$.

The KL divergence term can be thought to enforce a standard normal Bayesian prior on the latent space, where λ represents the strength of the prior. Thus, the KL divergence term allows for regularization of the latent space as well as encourages removal of information that is unnecessary for reconstruction from the latent space. In the DeepComBat CVAE, since biological and batch covariates are explicitly given to the decoder, optimal latent space representations should contain no information about these covariates and instead encode richer, subject‐specific information. Practically, this complete independence may be unrealistic to achieve, and this is accounted for during the harmonization step.

Importantly, while biological and batch covariates are used as inputs for both the encoder and the decoder, the CVAE is not rewarded for including information about these covariates in the loss function. This design choice prevents the CVAE from introducing bias, but still allows the model to learn multivariate batch effects conditional on potential biological confounders.

#### 2.3.4 Optimization and hyperparameter tuning

This CVAE loss function is known to have the potential to suffer from KL vanishing, also referred to as posterior collapse, where a local minimum of the loss function is reached and the model cannot improve (Bowman et al., 2016). In KL vanishing, the encoder learns to collapse all latent space representations to the standard normal prior such that the KL component of the loss function is nearly zero, and the decoder is given total noise and is therefore unable to learn anything in order to make progress toward further minimizing the loss. To minimize risk of posterior collapse in the DeepComBat CVAE, we utilize a cyclic annealing optimization schedule (Fu et al., 2019). In this schedule, λ is gradually increased from 0 to the goal final KL divergence weight multiple times over the course of model training. This provides opportunities for the optimizer to escape local minimum when λ is small and allows for progressive learning of more meaningful latent representations across cycles.

In DeepComBat, we perform manual hyperparameter tuning to determine our desired final $\lambda_{\text{Final}}=0.1$. The goal in tuning $\lambda_{\text{Final}}$ is to impose a prior that is strong enough to regularize the latent space and Euclidean distance between latent space representations are meaningful, but weak enough to allow for rich, subject‐specific information to be encoded in the latent space in order to produce high‐quality reconstructions.

Using this $\lambda_{\text{Final}}$, we first pre‐train the CVAE for 5 epochs with λ=0, then perform cyclic annealing over 30 epochs where one cycle is 5 epochs and λ increases linearly from 0 to $\lambda_{\text{Final}}$ within each cycle, and finally train the CVAE for 5 epochs with the desired $\lambda =\lambda_{\text{Final}}$. This cyclic annealing procedure is computationally helpful to improve convergence and is performed in a fully automated manner—users are only required to specify the desired $\lambda_{\text{Final}}$. Compared to a more straightforward constant‐schedule training schedule where the CVAE is trained using a constant $\lambda =\lambda_{\text{Final}}$ for all 40 total epochs, the cyclic annealing schedule leads to significantly lower final overall and reconstruction losses as well as significantly higher final prior losses when $\lambda_{\text{Final}}$ is large (Supplemental Figure 1, *p* < .05). Thus, cyclic schedule training may allow for better estimation of subject‐specific multivariate means using subject‐specific latent space factors that are independent of batch and biological covariates. Additionally, when $\lambda_{\text{Final}}$ is large, the variances of final reconstruction and prior losses appear qualitatively larger under the constant schedule compared to the cyclic schedule. This suggests cyclic schedule training may be more consistent compared to constant schedule training when trade‐offs between reconstruction loss and prior loss are high. We present results using cyclic annealing in the main text and provide additional results using both cyclic annealing and constant scheduling for various $\lambda_{\text{Final}}$ in the Supplemental Materials.

For internal DeepComBat, optimization was performed using the Adam optimizer with learning rate of 0.01, chosen to increase the initial rate of model convergence (Kingma & Ba, 2017). For external DeepComBat, optimization was performed using the AdamW optimizer, a version of Adam which shrinks parameter estimates toward 0 and therefore introduces regularization and reduces overfitting (Loshchilov & Hutter, 2019). For AdamW, we used a learning rate of 0.01 and default weight decay. Within epochs, data were passed to the CVAE in mini‐batches of 64 subjects.

Note that, in contrast to similar CVAE‐based harmonization methods like dcVAE, gcVAE, and a number of image‐based methods which require a KL divergence component hyperparameter such that latent space distributions are independent of batch, the DeepComBat $\lambda_{\text{Final}}$ is instead only used to regularize the latent space and reduce the amount of batch information in the latent space, if possible. However, substantial remaining batch information in the DeepComBat latent space is allowed, which enables easier hyperparameter tuning.

Additionally, in settings with larger numbers of features, such as with functional connectivity measures, the DeepComBat package allows for user specification of number of hidden layers as well as hidden layer and latent space sizes. Decreasing these hyperparameters will decrease the computational time necessary to train DeepComBat; however, other than to improve computational efficiency, these hyperparameters do not need to be tuned. Empirically, in the ADNI dataset, DeepComBat performance was robust to such changes in hyperparameters.

#### 2.3.5 Harmonization

Once the CVAE model has been trained, harmonization can be performed on the latent space, the CVAE decoder, and the reconstruction residuals, as shown in Figure 1. In the latent space, each subject's noisy latent space distribution, $Z_{\mathrm{ij}}$, is converted to the noiseless latent space mean vector, $\mu_{\mathrm{ij}}$. Then, across all ij subjects, the ComBat model described above is fitted using both batch and biological covariates to harmonize the latent space. Let each ComBat‐harmonized latent space representation be denoted as: $\mu_{\mathrm{ij}}^{\text{ComBat}}$.

Next, the decoder output is harmonized. In this step, the decoder input is changed such that it receives harmonized latent space mean vectors as well as the desired batch for the harmonized data. The decoder additionally continues to receive unchanged biological covariates.

Then, the reconstruction residuals are calculated and harmonized. To estimate these residuals, noiseless reconstructions are first estimated by giving the decoder latent space mean vectors instead of the latent space distribution samples used during CVAE training. Then, reconstruction residuals are defined as the difference between reconstructions and the original data. These residuals are then corrected across all subjects using the ComBat model with both batch and biological covariates.

#### 2.3.6 Speed

While DeepComBat is able to train quickly on a single CPU core on standard computers, including laptops, DeepComBat is still much slower than statistical methods. The overall training time is further increased when manual hyperparameter tuning is taken into consideration, as end‐users may need to train a few models before choosing a suitable $\lambda_{\text{Final}}$. Overall, hyperparameter tuning and final model training should take no longer than 5–10 min, depending on the number of hyperparameters tried and dataset size.

#### 2.3.7 Data and code availability

Data used in the preparation of this article were obtained from the ADNI database (adni.loni.usc.edu). The ADNI was launched in 2003 as a public‐private partnership, led by Principal Investigator Michael W. Weiner, MD. The primary goal of ADNI has been to test whether serial MRI, positron emission tomography (PET), other biological markers, and clinical and neuropsychological assessment can be combined to measure the progression of MCI and early AD. For up‐to‐date information, see www.adni-info.org.

An R package for performing DeepComBat is available at https://github.com/hufengling/DeepComBat. This package is written in ‘torch for R' which is the R analog of PyTorch that interfaces with the same C++ backend for fast computation. Across 30 runs, one full run of the DeepComBat algorithm on the ADNI dataset took an average of 53.0 s with standard deviation of 1.7 s on an Intel Xeon CPU with 2.40 GHz clock rate. Additionally, all code for evaluation and analysis is available at https://github.com/hufengling/deepcombat_analyses. Code for processing ADNI data is available at https://github.com/ntustison/CrossLong.

### 2.4 Evaluation

DeepComBat was evaluated against unharmonized data as well as other feature‐level harmonization methods where code was available. These methods included ComBat and CovBat in the main manuscript, and ComBat, CovBat, dcVAE, and gcVAE in the Supplemental Materials (An et al., 2022; Chen et al., 2022; Fortin et al., 2017; Moyer et al., 2020). Notably, since no code was provided in the original manuscript for dcVAE, we implemented this method using code provided by An et al. (2022). For all comparison methods, we used default settings and hyperparameters provided in the code. Biological covariates of age, sex, and AD status were provided for ComBat, CovBat, and DeepComBat. Evaluation was conducted using qualitative visualization, statistical testing, and ML experiments.

In statistical testing and ML experiments, we assess the presence of both batch effects and biological effects. When assessing for batch effects, we assume that (1) test statistics corresponding to large *p*‐values for statistical tests and (2) worse performance in predicting batch for ML experiments correspond to less presence of batch effects and therefore better harmonization. However, when assessing for biological effects, effective harmonization may lead to better, worse, or similar results, depending on the underlying relationship between batch and biological covariates.

#### 2.4.1 Qualitative visualization

We visualize the overall multivariate distribution of unharmonized and harmonized feature matrices using Unifold Manifold Approximation and Projection (UMAP) and principal component analysis (PCA) (McInnes et al., 2020). UMAP was fit using the umap package in R with 20 neighbors, 100 epochs, and default settings otherwise. Points were displayed by batch status. PCA was fit on correlation matrices to account for differences in scale across features. For UMAP and PCA, arbitrary differences in sign due to model fitting were changed in order to improve direct comparability of these visualizations between methods. Additionally, we explore how harmonization methods act on a small random sample of features using bivariate density plots and plots of feature‐level changes after harmonization.

#### 2.4.2 Statistical testing

Harmonization methods were evaluated using mass univariate and multivariate statistical testing. For mass univariate testing, we performed two‐sample Anderson–Darling test on each feature, between pairwise batches, resulting in three *p*‐values per feature. Average *p*‐value across all features and pairwise batches, as well as its standard deviation is reported.

To test for differences in feature‐wise means across batch as well as assess for validity of downstream analyses on biological covariates, we performed linear regression on each feature, where each regression model included the batch covariate as well as biological covariates of age, sex, and AD status. Batch *p*‐values are calculated via likelihood ratio test. For each covariate, the average negative log 10 *p*‐value across all features as well as the standard deviation of these transformed *p*‐values is reported. Negative log 10 *p*‐values are used to better represent the distribution of *p*‐values very close to 0.

For multivariate statistical testing, we assess harmonization results parametrically as well as nonparametrically. For parametric testing, we use the multivariate analysis of variance (MANOVA) test, which tests for differences in multivariate conditional means. Our MANOVA model includes the batch covariate, age, sex, and AD status. We report the negative log 10 *p*‐value based on Pillai's trace test statistic, which has been shown to be more robust than other MANOVA test statistics (Olson, 1974).

For nonparametric multivariate testing, we use the k‐nearest‐neighbor batch‐effect test (kBET) metric with 500 repeats and default settings otherwise (Büttner et al., 2019). The kBET test is a nonparametric permutation‐based test developed and validated in the context of detecting batch effects in single‐cell RNA‐sequencing (scRNA‐seq) that (1) randomly samples a proportion of observations, (2) identifies each observation's k‐nearest neighbors (KNNs), (3) evaluates whether the local distribution of batch among each set of KNNs differs from the global distribution of batch, and (4) generates an overall kBET statistic evaluating whether the number of observations with large differences in local distribution of batch are greater than that expected.

#### 2.4.3 ML experiments

To evaluate how our method interacts with multivariate batch or biological effects, we train ML algorithms to predict covariate information using the harmonized feature matrix. Prediction models were independently trained to perform classification of batch, sex, and AD status, as well as regression of age. To perform the ML experiments, we use the caret package, version 6.0‐93, to train and assess a large battery of ML algorithms on each feature matrix using the repeated cross‐validation strategy, with 10 repeats of 10‐fold cross‐validation to estimate the out‐of‐sample predictive performance.

For two‐class classification sex, average area under the receiver operating characteristic curve (AUROC) across validation sets is reported. For three‐class classification of batch and AD, AUROC cannot be calculated, so average accuracy across validation sets is reported. For regression of age, average $R^{2}$ values across validation sets is reported. Note that in the repeated cross‐validation strategy, average cross‐validation metrics can be made arbitrarily precise by increasing the number of repeats, but variation in these metrics occurs within each cross‐validation fold due to randomness in train‐validation splitting and ML model fitting.

The ML evaluation battery for classification tasks consisted of: support vector machine (SVM) with radial basis, quadratic discriminant analysis (QDA), KNNs, random forest (RF), and Extreme Gradient Boosted trees (XGBoost). The ML evaluation battery for regression of age consisted of: SVM with radial basis, KNN, RF, and XGBoost. SVM, QDA, KNN, and RF were fit using the default hyperparameters provided by their corresponding R packages. For XGBoost a few hyperparameters were a priori changed from the default to allow for greater algorithm differences when compared to RF. These changed hyperparameters included: eta = 0.1 and colsample_bytree = 0.5. Number of total boosting rounds had no default and was set to 100. Other hyperparameters were set to their defaults.

## 3 RESULTS

### 3.1 DeepComBat reduces batch effects in qualitative visualizations

**FIGURE 2:**
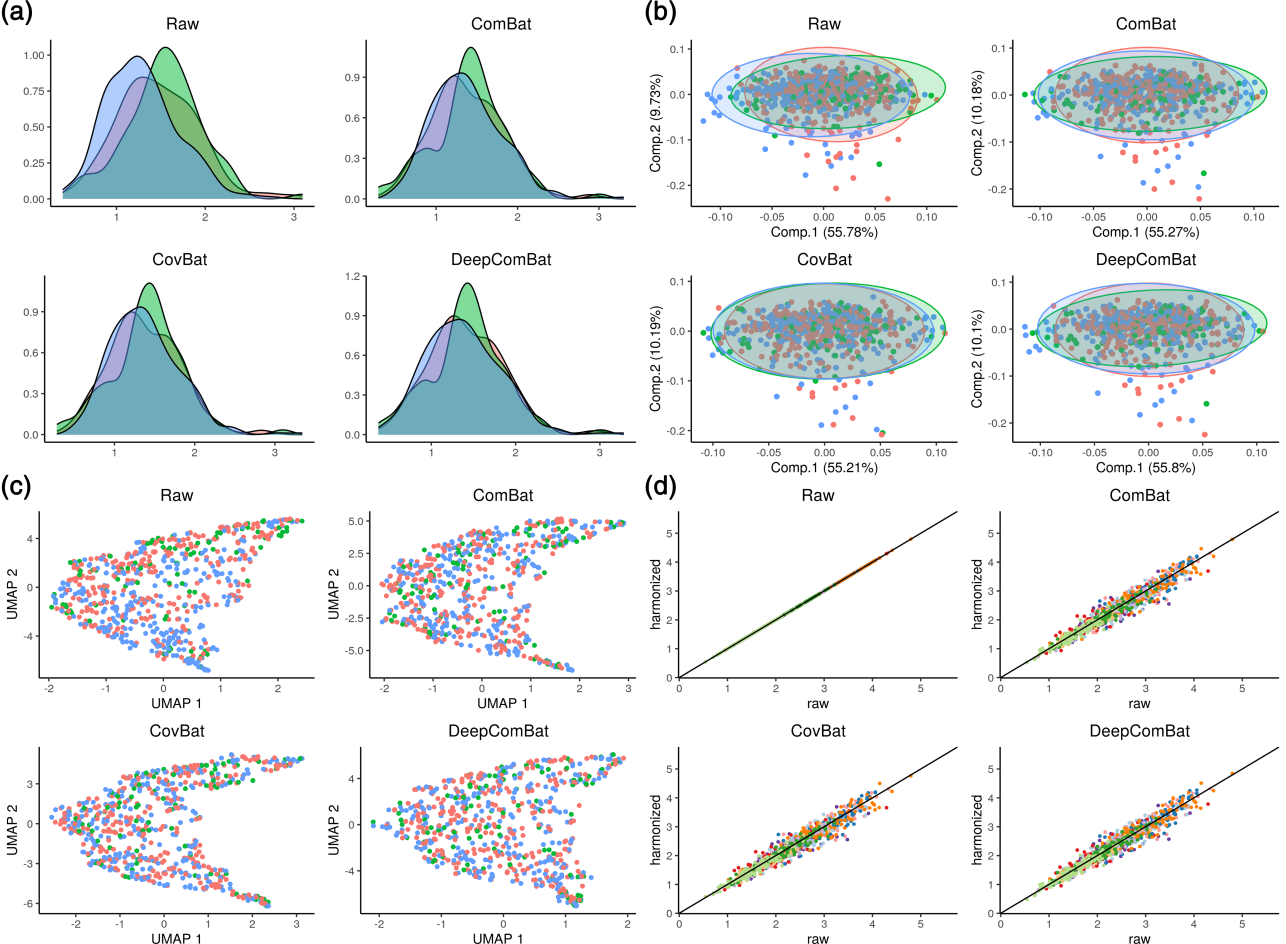
Internal harmonization qualitative visualizations. In panels a–c, red corresponds to Siemens, green corresponds to General Electric (GE), and blue corresponds to Philips. (a) Density plots of one randomly sampled feature. (b) Principal component analysis (PCA) plots where PCA ellipses denote major and minor axes for each batch, centered at the batch‐wise mean. (c) Unifold Manifold Approximation and Projection (UMAP) plots. (d) Randomly sampled harmonized values plotted against their corresponding raw values. Colors indicate each of the 10 randomly sampled cortical thickness features.

**FIGURE 3:**
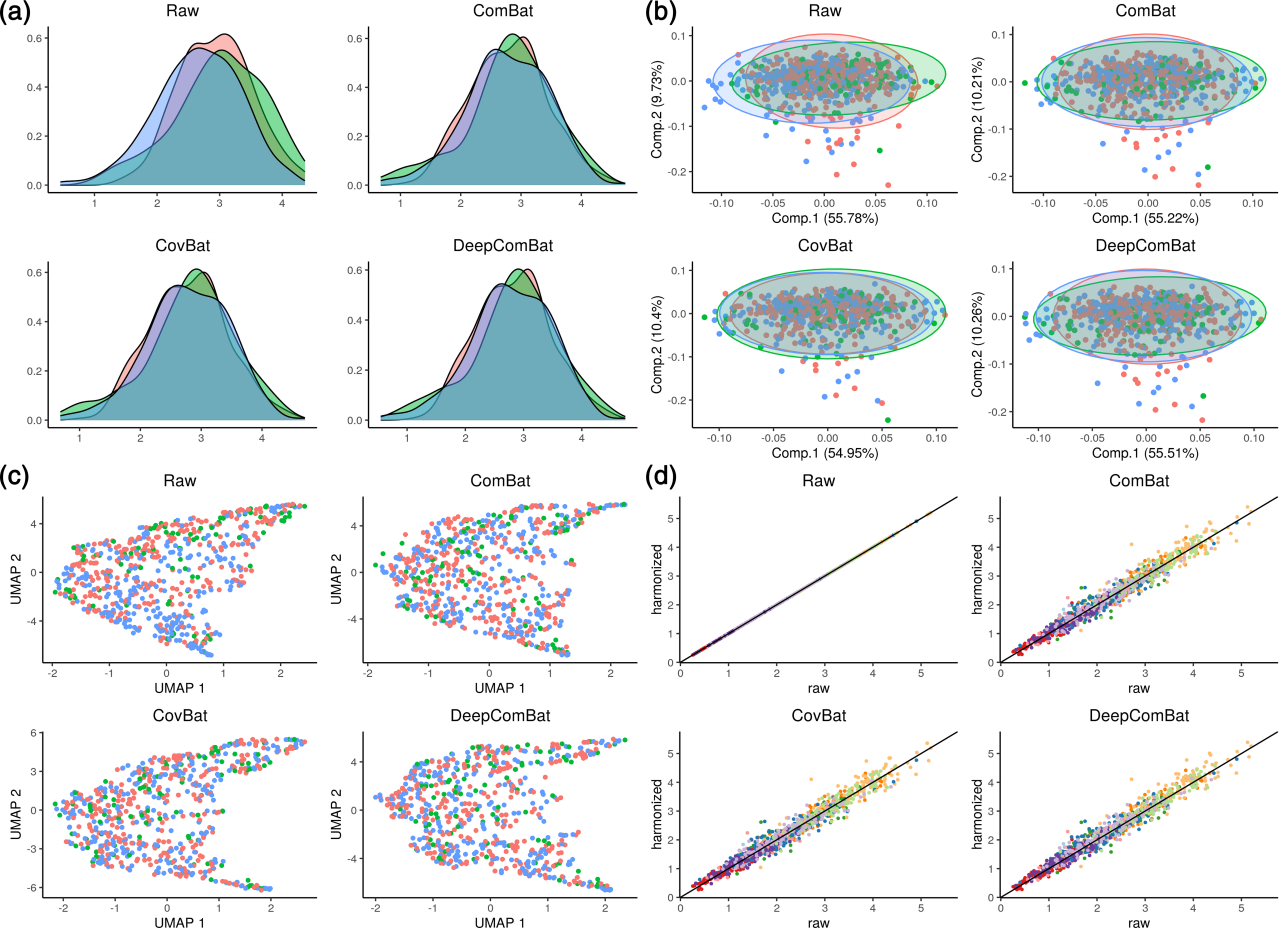
External harmonization qualitative visualizations. In panels a–c, red corresponds to Siemens, green corresponds to General Electric (GE), and blue corresponds to Philips. (a) Density plots of one randomly sampled feature. (b) Principal component analysis (PCA) plots where PCA ellipses denote major and minor axes for each batch, centered at the batch‐wise mean. (c) Unifold Manifold Approximation and Projection (UMAP) plots. (d) Randomly sampled harmonized values plotted against their corresponding raw values. Colors indicate each of the 10 randomly sampled cortical thickness features.

We visualized the effect of DeepComBat univariately and multivariately for internal and external DeepComBat. For a representative, randomly sampled region's cortical thickness, density plots by batch revealed differences in distribution across batch in the raw data that could be attributed to differences in mean, variance, and shape (Figures 2a and 3a). This distributional difference was qualitatively mitigated by ComBat, CovBat, and DeepComBat. Similar results were observed in visualizations of the multivariate feature distribution using the first two principal components and the first two UMAP dimensions (Figures 2b,c and 3b,c).

Finally, we explored how various harmonization methods change the raw data at the feature level. Here, we randomly sampled 10 cortical thickness features and randomly sampled 100 subjects to obtain a total of 1000 randomly sampled cortical thickness values. For each harmonization method, we plotted harmonized values for these cortical thicknesses against their corresponding raw values in Figures 2d and 3d. In this visualization, ComBat and CovBat seemed to mostly induce linear shifts in the data, consistent with their underlying shift and scale models. DeepComBat induced small nonlinear shifts in the data on a similar scale as ComBat and CovBat.

ComBat, CovBat, and DeepComBat performed similarly in the two‐batch setting, while dcVAE and gcVAE showed substantial transformation of the raw data (Supplemental Figure 2). Additionally, dcVAE and gcVAE mapped harmonized values to their corresponding CVAE‐predicted mean values without accounting for unmodeled CVAE reconstruction errors (Supplemental Figure 2d). Thus, dcVAE and gcVAE produced outputs with noise patterns characteristic of synthetic data, as noted by Dewey et al. (2019).

Overall, qualitative visualizations showed DeepComBat effectively matched univariate feature distributions across batches, preserved the underlying multivariate structure of the data, estimated harmonized values that were highly correlated with the corresponding raw values, and avoided introduction of synthetic artifacts.

### 3.2 DeepComBat removes statistically detectable batch effects and preserves inference on biological effects

Harmonization performance was assessed using parametric and nonparametric testing. Feature‐wise linear regression results are presented in Table 2 as average negative log 10 *p*‐values and in Supplemental Figures 3 and 4 as quantile–quantile plots of negative log 10 *p*‐value distributions. On the raw data, this analysis showed significant differences in mean across batch, when biological covariates were included in the model. These differences were effectively removed by ComBat, CovBat, and DeepComBat. In statistical testing for multivariate mean effects using MANOVA, ComBat, CovBat, and DeepComBat, completely removed batch effects from the multivariate mean across features when biological covariates were also included (Table 2).

**TABLE 2:** Parametric statistical testing results, reported as negative log 10 *p*‐values. Negative log 10 of conventional *p*‐value threshold 0.05 is 1.30. Larger is more significant.

**Internal harmonization**					
**Linear regression**—Mean (SD)					
	Batch	Age	Sex	AD Status (CN)	AD Status (LMCI)
Raw	8.7 (7.82)	14.91 (6.16)	0.82 (0.75)	15.47 (7.75)	6.06 (3.13)
ComBat	0.11 (0.09)	14.71 (6.21)	0.82 (0.75)	15.86 (8.32)	6.24 (3.13)
CovBat	0.01 (0.01)	14.57 (6.22)	0.82 (0.75)	15.69 (8.36)	6.19 (3.16)
DeepComBat	0.13 (0.11)	14.82 (6.25)	0.82 (0.77)	15.94 (8.41)	6.29 (3.20)
**Multivariate analysis of variance**					
	Batch	Age	Sex	AD Status	
Raw	89.18	33.55	25.46	15.56	
ComBat	0	32.41	17.64	17.10	
CovBat	0	32.40	17.12	17.34	
DeepComBat	0	32.82	17.77	17.52	
**External harmonization**					
**Linear regression**—Mean (SD)					
	Batch	Age	Sex	AD Status (CN)	AD Status (LMCI)
Raw	8.7 (7.82)	14.91 (6.16)	0.82 (0.75)	15.47 (7.74)	6.06 (3.13)
ComBat	0.12 (0.10)	14.58 (6.16)	0.82 (0.75)	15.96 (8.07)	6.24 (3.12)
CovBat	0.01 (0.01)	14.37 (6.15)	0.82 (0.75)	15.68 (8.06)	6.16 (3.15)
DeepComBat	0.10 (0.06)	14.74 (6.18)	0.82 (0.75)	16.16 (8.26)	6.30 (3.19)
**Multivariate analysis of variance**					
	Batch	Age	Sex	AD Status	
Raw	89.18	33.55	25.46	15.56	
ComBat	0	31.86	17.46	16.87	
CovBat	0	31.69	16.68	16.84	
DeepComBat	0	31.74	17.18	17.12	

In both univariate and multivariate analyses, ComBat, CovBat, and DeepComBat preserved inference on biological covariates of age, sex, and AD status. Notably, DeepComBat was slightly more powerful than ComBat and CovBat for detecting almost all biological covariate effects in both internal and external harmonization settings. These increases in power for inference may reflect removal of batch‐attributable confounding.

In nonparametric testing, average feature‐wise Anderson–Darling test results showed significant differences in univariate distributions across batches in the raw data (Table 3). These differences were effectively reduced by ComBat, CovBat, and DeepComBat. These results are further illustrated in Supplemental Figures 3 and 4. In these figures, ComBat, CovBat, and DeepComBat show *p*‐value distributions qualitatively similar to a uniform distribution, while raw data showed *p*‐value distributions with more highly significant *p*‐values than expected under a uniform distribution. Finally, when assessed via kBET, CovBat and DeepComBat both produced harmonized outputs where the distributions of batch within local neighborhoods were not significantly different from the global distribution, though DeepComBat showed a slight advantage in both internal and external harmonization (Table 3). In contrast, kBET detected highly significant differences for raw data and ComBat.

**TABLE 3:** Nonparametric statistical testing results. Higher *p*‐value corresponds to less statistically detectable differences between batches.

	Anderson–Darling	kBET
*Internal harmonization*		
Raw	0.13 (0.24)	0
ComBat	0.45 (0.29)	<0.001
CovBat	0.51 (0.26)	0.625
DeepComBat	0.45 (0.25)	0.664
*External harmonization*		
Raw	0.13 (0.24)	0
ComBat	0.45 (0.29)	<0.001
CovBat	0.50 (0.26)	0.559
DeepComBat	0.42 (0.23)	0.821

In the two‐batch setting, dcVAE and gcVAE increased the statistical difference in batch‐wise univariate means and did not eliminate differences in multivariate means (Supplemental Table 1). Meanwhile, dcVAE and gcVAE showed large increases in univariate power for age and AD effects and a large decrease in power for sex effects. These patterns may be explained by the exclusion of unmodeled residuals in dcVAE and gcVAE harmonized outputs, since statistical power is a function of both the effect size and the variance of residuals. dcVAE and gcVAE also performed poorly in nonparametric testing, according to both Anderson–Darling tests and kBET (Supplemental Table 2). These findings are qualitatively supported in Supplemental Figure 5.

Overall, DeepComBat removed statistically detectable batch effects and preserved biological information without introducing detectable bias. In univariate analyses, DeepComBat performed similarly to ComBat and CovBat, while in multivariate analyses, DeepComBat outperformed ComBat and CovBat. DeepComBat outperformed dcVAE and gcVAE by all metrics.

### 3.3 DeepComBat impairs detection of batch by ML algorithms and maintains predictability of biological covariates

**FIGURE 4:**
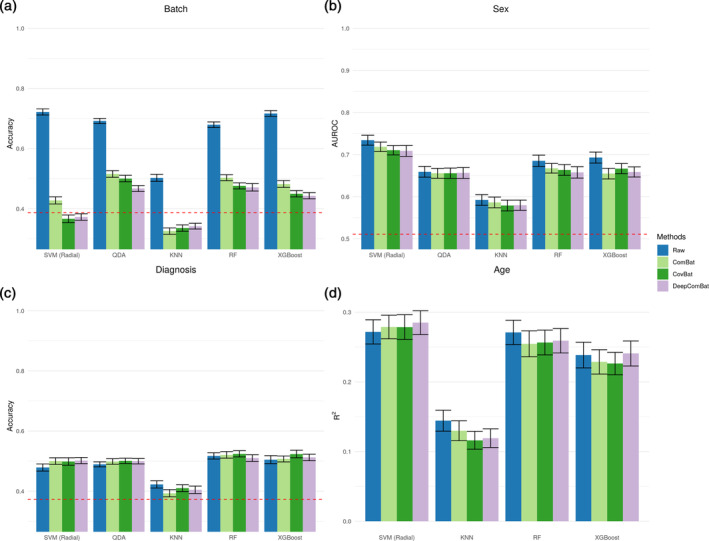
Internal harmonization machine learning results. Validation set performance metrics are shown for 10 repeats of 10‐fold cross validation. Error bars correspond to 95% confidence intervals. Dashed red lines display expected performance of a weighted random classifier. (a) Average accuracy for predicting batch. Lower is better. (b) Average area under the receiver operating characteristic curve (AUROC) for predicting sex. (c) Average accuracy for predicting Alzheimer disease status. (d) Average $R^{2}$ value for predicting age.

**FIGURE 5:**
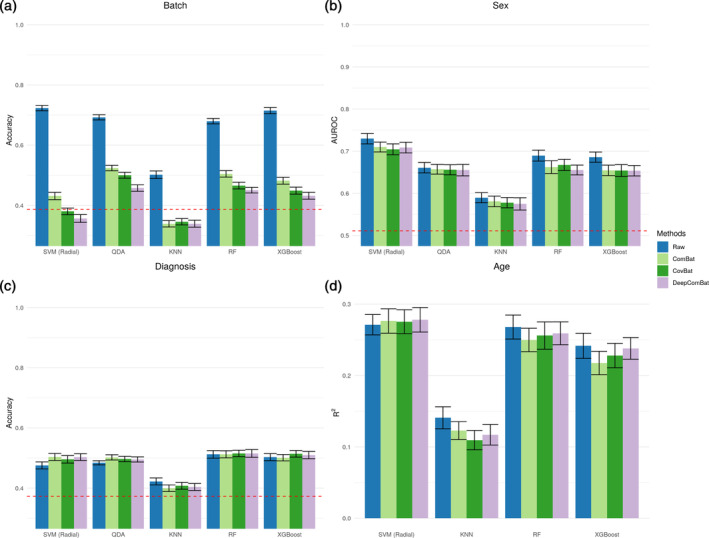
External harmonization machine learning results. Validation set performance metrics are shown for 10 repeats of 10‐fold cross validation. Error bars correspond to 95% confidence intervals. Dashed red lines display expected performance of a weighted random classifier. (a) Average accuracy for predicting batch. Lower is better. (b) Average area under the receiver operating characteristic curve (AUROC) for predicting sex. (c) Average accuracy for predicting Alzheimer disease status. (d) Average $R^{2}$ value for predicting age.

A battery of ML experiments seeking to predict batch status were run on raw and harmonized data (Figures 4a and 5a). All classifiers could effectively determine the batch status of out‐of‐sample subjects in the raw data. This ability to detect batch was greatly decreased by all harmonization methods, with DeepComBat‐harmonized data consistently corresponding to the lowest mean accuracy, especially for the ML algorithms with overall higher ability to discriminate batch.

Additionally, DeepComBat effectively retained biological information in its outputs. In Figure 4 and 5, DeepComBat‐harmonized data showed predictive performances similar to those of raw, ComBat‐corrected, and CovBat‐corrected data. In the case of age, removal of batch effects using DeepComBat increased the detectability of age‐related information in the resulting data.

In two‐batch harmonization, DeepComBat‐harmonized data contained less batch information that data harmonized by other methods, and dcVAE and gcVAE removed less batch effects compared to ComBat, CovBat, and DeepComBat (Supplemental Figure 6). All post‐harmonization predictive performances for biological covariates under ComBat, CovBat, and DeepComBat were significantly higher than those of dcVAE and gcVAE, suggesting that ignoring CVAE reconstruction residuals may be detrimental to preservation of non‐batch information.

Overall, we found that DeepComBat more effectively removed multivariate batch effects than other harmonization methods, even when assessed via powerful ML algorithms such as XGBoost. Additionally, DeepComBat effectively preserved biological information in the predictive context.

### 3.4 DeepComBat is robust to hyperparameter choice and training schedule

Empirically, DeepComBat still performed effective harmonization over a range of $\lambda_{\text{Final}}$ and using either cyclic annealing or constant training schedule. The above analyses were run on DeepComBat internal harmonization outputs under a wide range of hyperparameter values. These values ranged from 16 times greater than the $\lambda_{\text{Final}}$ used in our primary analysis to values 16 times less. Loss profiles for these robustness analyses are presented in Supplemental Figure 1. Harmonization results from these analyses are presented in Supplemental Tables 3 and 4 and Supplemental Figures 7–9. Overall, results for the highest value of $\lambda_{\text{Final}}$, tuning using either cyclic annealing or constant scheduling, showed superior harmonization according to ML experiments and similar performance according to statistical tests.

This robustness result may be due to the design of DeepComBat, which partitions batch effects originally present in the raw data into one of these three components: the CVAE latent space, the CVAE decoder, and the reconstructed residuals. Component‐wise harmonization therefore allows for a form of “double‐robustness” with respect to CVAE fitting—if $\lambda_{\text{Final}}$ is too large such that most batch effects are contained in the reconstruction residuals, ComBat on these residuals will still allow for reasonable overall harmonization; and if $\lambda_{\text{Final}}$ is too small such that most batch effects are contained in the latent space, ComBat on the latent space will address the batch effects.

## 4 DISCUSSION

Multi‐batch neuroimaging data are increasingly common and necessary for learning generalizable models for inference and prediction. There is also growing interest in using ML techniques to perform multivariate pattern analysis and train powerful classifiers that can efficiently use multivariate data. To enable these efforts, there is increasing need for statistically rigorous multivariate harmonization methods.

In this study, we demonstrate that strong batch effects exist in raw data, and that these batch effects remain detectable by ML experiments even after state‐of‐the‐art statistical harmonization methods are applied. We also find that, while previously proposed deep learning harmonization approaches are able to partially remove batch effects from the ADNI dataset, this batch effects correction comes at the cost of removal of relevant biological information as well as introduction of artifacts characteristic of synthetic data. We then propose DeepComBat, a novel hybrid method that is able to take advantage of the strengths of both deep learning and statistical methods—it uses the CVAE architecture to perform nonlinear, multivariate correction as well as the ComBat model to rigorously and robustly harmonize the latent space and residuals.

### 4.1 DeepComBat performance

When compared to other methods, we show DeepComBat performs more effectively when evaluated by highly multivariate ML experiments as well as nonparametric kBET testing. It performs comparably to ComBat and CovBat when evaluated by statistical tests. Meanwhile, DeepComBat had comparable predictive performance for sex, diagnosis, and age when compared to ComBat and CovBat, but superior performance when compared to dcVAE and gcVAE in the two‐batch setting. While increased biological effect sizes after harmonization may be desirable, the way in which biological effect sizes change after harmonization are known to depend on the ground truth magnitude of biological and batch effects, as well as on the nature of confounding between batch and biology (Hu et al., 2023).

For example, in this dataset, sex is observed to be imbalanced across the batches such that knowing a subject's batch provides information regarding the subject's sex. In this setting, if the ground truth sex effect is small relative to the batch effect, a classifier for sex trained on harmonized data should theoretically perform worse than the same classifier trained on raw data, and this pattern is empirically observed for ComBat, CovBat, and DeepComBat in Table 2 and Figures 4b and 5b. However, since no ground truth for post‐harmonization sex effect magnitude exists, it is unclear how much reduction in predictive performance for sex is accurate and therefore which method best recovers the ground truth. Similarly, an increase in estimates of biological effects may occur if minimal imbalance is present, but batch effect introduces bias or noise that covers up a smaller biological signal. This may occur in settings where one batch produces features of much higher quality than another, such as if one batch corresponds to higher Tesla imaging compared to another. Finally, no change in estimated biological effects may be expected if neither situation occurs or if both occur but balance each other out. However, regardless of how estimated biological effects change after harmonization, improved removal of batch effects leads to more generalizable and reproducible inference, since results can be trusted to be free of complex batch‐related confounding structures.

Additionally, we empirically find that, in our dataset, higher values of $\lambda_{\text{Final}}$ lead to slightly superior DeepComBat performance under both cyclic annealing and constant scheduling. This suggests more extensive tuning of $\lambda_{\text{Final}}$ for each new dataset could lead to superior results; however, increasing $\lambda_{\text{Final}}$ by too much will lead to elimination of all subject‐specific information from the CVAE latent space and forego the benefits of DeepComBat over standard ComBat. Thus, we choose to use a relatively small $\lambda_{\text{Final}}$ of 0.1 by default. This choice allows for adequate latent space regularization with minimal risk of overweighting the prior loss. Similarly, we fit DeepComBat using cyclic annealing by default despite comparable performance under constant scheduling—since cyclic annealing allows for better stability with respect to KL vanishing when $\lambda_{\text{Final}}$ is high, cyclic annealing may provide better fitting in certain datasets. To provide flexibility, we provide functionality for manual selection of $\lambda_{\text{Final}}$ as well as training schedule in the DeepComBat R package.

Overall, these results suggest that DeepComBat may be especially useful for harmonization in settings where prediction or inference using multivariate features and multivariate methods is the goal. In these settings, feature‐wise correction using statistical methods may lead to significant non‐corrected batch effects that may be picked up by prediction methods and inappropriately used.

### 4.2 DeepComBat may be more robust to model misspecification when compared to statistical methods

Similarly to many statistical methods, such as ComBat and CovBat, DeepComBat assumes batch effects can be estimated as differences in feature‐wise conditional means and variances of unmodeled residuals. However, unlike statistical methods, mean batch effect are estimated nonlinearly and multivariately using a combination of batch and biological covariates along with subject‐specific latent space representations. In this mean batch effect estimation procedure, mean batch effect are partially removed by the decoder in a nonparametric manner, where the only assumption on the nature of batch effect is that it can be approximated by the decoder network. Thus, while latent space harmonization still involves the ComBat model, overall harmonization may be less contingent on how well the data follow ComBat assumptions. Additionally, discussed further below, moment‐matching of latent space representations has been empirically shown to be effective in various harmonization‐like tasks (Fatania et al., 2022; Huang & Belongie, 2017; Lopez et al., 2018; Lotfollahi et al., 2019; Zuo et al., 2021).

In terms of correcting batch effect in unmodeled residuals, DeepComBat argues a meaningful portion of what statistical methods claims are “unmodeled residuals”—information that is not explained by biological nor batch covariates by the naive linear model—can in fact be explained as a multivariate nonlinear function of biological covariates, batch covariates, and subject‐specific latent factors. Through the CVAE architecture, DeepComBat is able to significantly reduce the mean squared error between model‐predicted feature vectors and raw feature vectors when compared to ComBat and CovBat. Thus, DeepComBat is able to directly model and correct more batch effect in terms of conditional differences in mean, and less batch effect is corrected based on the strong assumption that there are batch‐wise differences in the variances of unmodeled residuals. Subsequently, although DeepComBat still uses the ComBat model to correct the residuals, it may rely less on ComBat‐specific assumptions since the magnitude of batch effect correction on the residuals is smaller.

### 4.3 DeepComBat relaxes strong assumptions made by other deep learning methods and simplifies model fitting

Previous feature‐level deep learning harmonization methods, including dcVAE and gcVAE make a number of strong implicit assumptions. These assumptions include (1) perfect model fit, which assumes that reconstruction residuals insignificant and therefore do not need to accounted for or reincorporated, (2) fully disentangleable latent space, which assumes that the neural network can completely learn a batch‐invariant latent space based on the loss function alone, and (3) balanced biological covariates across batches, which assumes that all population‐level differences across batch are in fact due to batch and should be removed.

However, the first assumption is violated in situations where the latent‐space dimensions are too small to adequately capture non‐batch information, the decoder is not complex enough to efficiently encode all batch‐related information, and sample sizes within batches are too limited to estimate all the necessary network parameters. These violations are further compounded when the first two assumptions are considered together, while near‐perfect model fit may be achievable with a large latent space, it is even more challenging when a completely batch‐invariant latent space is required. Finally, in neuroimaging datasets where biological covariates are imbalanced across batches, such as in the ADNI dataset used in this study, complete removal of marginal batch‐wise differences will necessarily involve removal of biological information as well.

DeepComBat is able to relax these strong implicit assumptions by (1) accounting for the presence of reconstruction residuals and reintroducing them on top of the CVAE‐harmonized subject‐level means, (2) explicitly removing batch effects from the CVAE latent space, and (3) conditioning on biological covariates at each harmonization step. By relaxing these assumptions, we are able to greatly improve the usability of DeepComBat by simplifying its architecture when compared to that of dcVAE and gcVAE. For example, dcVAE and gcVAE rely on adversarial training with a discriminator in order to train their decoders to produce more realistic outputs, but DeepComBat no longer needs this adversarial component since non‐perfect model fit is acceptable. This minimizes computational burden and avoids common challenges in adversarial training. DeepComBat also circumvents the need for precise tuning of the KL divergence weighting hyperparameter, since remaining batch effects in the latent space are explicitly removed after CVAE training.

Importantly, relaxing these assumptions allows for DeepComBat to be designed such that, if a subject‐level feature vector is “self‐harmonized” back to its actual batch, that feature vector will be unchanged. This makes sense, since “self‐harmonization” should be the identity function. However, in other deep learning harmonization methods, including dcVAE and gcVAE, since reconstruction residuals are not explicitly accounted for in these other methods, the “self‐harmonized” data will have less noise. This phenomena has been highlighted by Dewey et al. (2019), who noticed that DeepHarmony, an image‐level harmonization method, produced harmonized images with noise characteristics indicative of a synthetic image—namely, that they looked smoother. By keeping reconstruction residuals in the final harmonized output, DeepComBat avoids an implicit assumption of perfect model fit and allows for harmonized outputs to retain natural noise characteristics.

### 4.4 DeepComBat resembles other moment‐matching harmonization methods

DeepComBat primarily achieves multivariate harmonization by using ComBat in the CVAE latent space in order to generate a batch‐invariant latent space. In this step, ComBat is used as a moment‐matching model that takes advantage of shrinkage estimation in order to match conditional means and variances across batches. Analogies between latent‐space ComBat and other moment‐matching style transfer algorithms can be drawn.

Specifically, in scRNA‐seq batch effects correction, scGen encodes gene expression data to a latent space using a standard variational autoencoder (Lotfollahi et al., 2019). Then, the algorithm estimates and removes mean batch effects, or first moments, from this latent space, conditional on cell type, in order to perform correction. CVAE‐based methods such as dcVAE, gcVAE, and a number of scRNA‐seq methods, such as scVI, can also be thought to perform latent‐space moment‐matching (Lopez et al., 2018); however, these methods do so implicitly through the loss function, rather than by explicitly estimating and correcting latent space coordinates.

Additionally, in image style transfer, where the goal is to change the style of an image without changing its content, adaptive instance normalization (AdaIN) can be used along with a convolutional autoencoder and its variations (Huang & Belongie, 2017). In the convolutional autoencoder, images are encoded into a set of latent space convolutional filters. Then, AdaIN performs style transfer by matching the means and variances of each filter, learned from the original image, to the means and variances of the corresponding filters learned from the image that has the desired style. In the non‐convolutional setting of DeepComBat, each 1×1 element of the latent space vector corresponds to one convolutional filter, and similar moment‐matching is performed, but at the group level instead of the individual input level.

Finally, outside of deep learning methods, DeepComBat draws on ideas from CovBat, which has been shown to harmonize the mean and covariance across sites. CovBat first performs standard ComBat and then corrects the covariance structure of residuals by projecting them into a latent space defined by principal components and running ComBat again. Thus, CovBat performs univariate mean harmonization and linearly multivariate residual harmonization. DeepComBat flips these steps—it first performs nonlinear multivariate mean harmonization and then univariately corrects the reconstruction residuals. Notably, DeepComBat autoencoder residuals are much smaller in magnitude than CovBat linear model residuals, so univariate residual correction is sufficient.

### 4.5 Limitations

We show DeepComBat to be a promising tool for multivariate, deep learning harmonization. However, more work must be done to continue characterizing and extending DeepComBat. First, DeepComBat performance should be validated in other forms of high‐dimensional, large neuroimaging datasets with highly correlated features. Examples of such datasets include voxel‐wise images, diffusion imaging features, as well as functional connectivity and other network features. Additionally, while DeepComBat is statistically rigorous and robust to hyperparameter choice, training schedules, and both internal and external harmonization settings, DeepComBat still contains black box deep learning elements which may limit utility in settings where traceability is essential, such as in clinical trials. Finally, DeepComBat is designed for harmonization of cross‐sectional studies where only one batch variable is present and cannot be applied to longitudinal datasets or datasets where two or more variables introduce technical noise. In clinical or non‐cross‐sectional datasets, well‐characterized methods with analytical solutions, such as standard ComBat and its variants, may be a more reasonable choice (Hu et al., 2023).

## 5 CONCLUSION

DeepComBat is a novel, statistically rigorous, deep learning approach to image harmonization that leverages deep learning and statistical concepts to perform multivariate batch effects correction conditional on biological covariates. We demonstrate it can more effectively remove multivariate batch effects from structural neuroimaging feature while preserving biological information than previously proposed methods. Additionally, DeepComBat proposes marked innovations over previous deep learning harmonization methods, allowing for conditioning on covariates, preservation of raw data characteristics, harmonization of more than two batches, robustness to choice of hyperparameters, and external harmonization capabilities. As high‐dimensional, multi‐batch data becomes more common and interest in using ML techniques to analyze such data grows, we hope that DeepComBat will serve as a tool for end‐users to remove multivariate batch confounding. Additionally, we hope the statistically motivated design of DeepComBat provides a new perspective for methodologists to continue developing improved deep learning harmonization methods.

## AUTHOR CONTRIBUTION STATEMENT

**Fengling Hu**: Conceptualization, methodology, software, validation, formal analysis, investigation, writing—original draft, writing—review and editing, visualization. **Alfredo Lucas**: Software, validation, investigation, visualization, writing—review and editing. **Andrew A. Chen**: Methodology, software, validation. **Kyle Coleman**: Methodology, software. **Hannah Horng**: Validation. **Raymond W.S. Ng**: Software, investigation. **Nicholas J. Tustison**: Resources, data curation. **Haochang Shou**: Methodology, investigation, writing—review and editing. **Kathryn A. Davis**: Resources, funding acquisition. **Mingyao Li**: Conceptualization, methodology, writing—review and editing, supervision, funding acquisition. **Russell T. Shinohara**: Conceptualization, methodology, investigation, resources, writing—review and editing, supervision, project administration, funding acquisition.

## FUNDING INFORMATION

This study was supported by grants from the National Institute of Neurological Disorders and Stroke (R01NS085211 and R01NS060910), the National Multiple Sclerosis Society (RG‐1707‐28586), and the University of Pennsylvania Center for Biomedical Image Computing and Analytics (CBICA). Funding sources were not involved in study design, data analysis, manuscript preparation, or submission decisions. Data collection and sharing for this project was funded by the Alzheimer's Disease Neuroimaging Initiative (ADNI) (National Institutes of Health Grant U01 AG024904) and DOD ADNI (Department of Defense award number W81XWH‐12‐2‐0012). ADNI is funded by the National Institute on Aging, the National Institute of Biomedical Imaging and Bioengineering, and through generous contributions from the following: AbbVie, Alzheimer's Association; Alzheimer's Drug Discovery Foundation; Araclon Biotech; BioClinica, Inc.; Biogen; Bristol‐Myers Squibb Company; CereSpir, Inc.; Cogstate; Eisai Inc.; Elan Pharmaceuticals, Inc.; Eli Lilly and Company; EuroImmun; F. Hoffmann‐La Roche Ltd and its affiliated company Genentech, Inc.; Fujirebio; GE Healthcare; IXICO Ltd.; Janssen Alzheimer Immunotherapy Research & Development, LLC.; Johnson & Johnson Pharmaceutical Research & Development LLC.; Lumosity; Lundbeck; Merck & Co., Inc.; Meso Scale Diagnostics, LLC.; NeuroRx Research; Neurotrack Technologies; Novartis Pharmaceuticals Corporation; Pfizer Inc.; Piramal Imaging; Servier; Takeda Pharmaceutical Company; and Transition Therapeutics. The Canadian Institutes of Health Research is providing funds to support ADNI clinical sites in Canada. Private sector contributions are facilitated by the Foundation for the National Institutes of Health (www.fnih.org). The grantee organization is the Northern California Institute for Research and Education, and the study is coordinated by the Alzheimer's Therapeutic Research Institute at the University of Southern California. ADNI data are disseminated by the Laboratory for Neuro Imaging at the University of Southern California.

## CONFLICT OF INTEREST STATEMENT

RTS receives consulting income from Octave Bioscience and compensation for reviewership duties from the American Medical Association. The authors report no conflicts of interest.

## Supporting information


**DATA S1:** Supporting Information.

## Data Availability

Data used in the preparation of this article were obtained from the Alzheimer's Disease Neuroimaging Initiative (ADNI) database (adni.loni.usc.edu). The ADNI was launched in 2003 as a public‐private partnership, led by Principal Investigator Michael W. Weiner, MD. The primary goal of ADNI has been to test whether serial MRI, PET, other biological markers, and clinical and neuropsychological assessment can be combined to measure the progression of MCI and early AD. For up‐to‐date information, see www.adni-info.org. An R package for performing DeepComBat is available at https://github.com/hufengling/DeepComBat. All code for evaluation and analysis is available at https://github.com/hufengling/deepcombat_analyses. Code for processing ADNI data are available at https://github.com/ntustison/CrossLong.

## References

[hbm26708-bib-0001] Acquitter, C. , Piram, L. , Sabatini, U. , Gilhodes, J. , Moyal Cohen‐Jonathan, E. , Ken, S. , & Lemasson, B. (2022). Radiomics‐based detection of radionecrosis using harmonized multiparametric MRI. Cancers, 14, 286. 10.3390/cancers14020286 35053450 PMC8773614

[hbm26708-bib-0002] An, L. , Chen, J. , Chen, P. , Zhang, C. , He, T. , Chen, C. , Zhou, J. H. , & Yeo, B. T. T. (2022). Goal‐specific brain MRI harmonization. NeuroImage, 263, 119570. 10.1016/j.neuroimage.2022.119570 35987490

[hbm26708-bib-0003] Avalos‐Pacheco, A. , Rossell, D. , & Savage, R. S. (2022). Heterogeneous large datasets integration using Bayesian factor regression. Bayesian Analysis, 17, 33–66. 10.1214/20-BA1240

[hbm26708-bib-0004] Avants, B. , Klein, A. , Tustison, N. , Woo, J. , & Gee, J. C. (2010). Evaluation of open‐access, automated brain extraction methods on multi‐site multi‐disorder data. In: 16th Annual Meeting for the Organization of Human Brain Mapping.

[hbm26708-bib-0005] Avants, B. B. , Tustison, N. J. , Wu, J. , Cook, P. A. , & Gee, J. C. (2011). An open source multivariate framework for n‐tissue segmentation with evaluation on public data. Neuroinformatics, 9, 381–400. 10.1007/s12021-011-9109-y 21373993 PMC3297199

[hbm26708-bib-0006] Badhwar, A. , Collin‐Verreault, Y. , Orban, P. , Urchs, S. , Chouinard, I. , Vogel, J. , Potvin, O. , Duchesne, S. , & Bellec, P. (2020). Multivariate consistency of resting‐state fMRI connectivity maps acquired on a single individual over 2.5 years, 13 sites and 3 vendors. NeuroImage, 205, 116210. 10.1016/j.neuroimage.2019.116210 31593793

[hbm26708-bib-0007] Bartlett, E. A. , DeLorenzo, C. , Sharma, P. , Yang, J. , Zhang, M. , Petkova, E. , Weissman, M. , McGrath, P. J. , Fava, M. , Ogden, R. T. , Kurian, B. T. , Malchow, A. , Cooper, C. M. , Trombello, J. M. , McInnis, M. , Adams, P. , Oquendo, M. A. , Pizzagalli, D. A. , Trivedi, M. , & Parsey, R. V. (2018). Pretreatment and early‐treatment cortical thickness is associated with SSRI treatment response in major depressive disorder. Neuropsychopharmacology, 43, 2221–2230. 10.1038/s41386-018-0122-9 29955151 PMC6135779

[hbm26708-bib-0008] Bayer, J. M. M. , Thompson, P. M. , Ching, C. R. K. , Liu, M. , Chen, A. , Panzenhagen, A. C. , Jahanshad, N. , Marquand, A. , Schmaal, L. , & Sämann, P. G. (2022). Site effects how‐to and when: An overview of retrospective techniques to accommodate site effects in multi‐site neuroimaging analyses. Frontiers in Neurology, 13, 923988. 10.3389/fneur.2022.923988 36388214 PMC9661923

[hbm26708-bib-0009] Bethlehem, R.a. I. , Seidlitz, J. , White, S. R. , Vogel, J. W. , Anderson, K. M. , Adamson, C. , Adler, S. , Alexopoulos, G. S. , Anagnostou, E. , Areces‐Gonzalez, A. , Astle, D. E. , Auyeung, B. , Ayub, M. , Bae, J. , Ball, G. , Baron‐Cohen, S. , Beare, R. , Bedford, S. A. , Benegal, V. , … Alexander‐Bloch, A. F. (2022). Brain charts for the human lifespan. Nature, 604, 525–533. 10.1038/s41586-022-04554-y 35388223 PMC9021021

[hbm26708-bib-0010] Bostami, B. , Hillary, F. G. , van der Horn, H. J. , van der Naalt, J. , Calhoun, V. D. , & Vergara, V. M. (2022). A decentralized ComBat algorithm and applications to functional network connectivity. Frontiers in Neurology, 13, 826734. 10.3389/fneur.2022.826734 35370895 PMC8965063

[hbm26708-bib-0011] Bourbonne, V. , Jaouen, V. , Nguyen, T. A. , Tissot, V. , Doucet, L. , Hatt, M. , Visvikis, D. , Pradier, O. , Valéri, A. , Fournier, G. , & Schick, U. (2021). Development of a radiomic‐based model predicting lymph node involvement in prostate cancer patients. Cancers, 13, 5672. 10.3390/cancers13225672 34830828 PMC8616049

[hbm26708-bib-0012] Bowman, S. R. , Vilnis, L. , Vinyals, O. , Dai, A. M. , Jozefowicz, R. , & Bengio, S. (2016). Generating sentences from a continuous space.

[hbm26708-bib-0013] Büttner, M. , Miao, Z. , Wolf, F. A. , Teichmann, S. A. , & Theis, F. J. (2019). A test metric for assessing single‐cell RNA‐seq batch correction. Nature Methods, 16, 43–49. 10.1038/s41592-018-0254-1 30573817

[hbm26708-bib-0014] Casey, B. J. , Cannonier, T. , Conley, M. I. , Cohen, A. O. , Barch, D. M. , Heitzeg, M. M. , Soules, M. E. , Teslovich, T. , Dellarco, D. V. , Garavan, H. , Orr, C. A. , Wager, T. D. , Banich, M. T. , Speer, N. K. , Sutherland, M. T. , Riedel, M. C. , Dick, A. S. , Bjork, J. M. , Thomas, K. M. , … Dale, A. M. (2018). The adolescent brain cognitive development (ABCD) study: Imaging acquisition across 21 sites. Developmental Cognitive Neuroscience, 32, 43–54. 10.1016/j.dcn.2018.03.001 29567376 PMC5999559

[hbm26708-bib-0015] Chen, A. A. , Beer, J. C. , Tustison, N. J. , Cook, P. A. , Shinohara, R. T. , Shou, H. , & Alzheimer's Disease Neuroimaging Initiative . (2022). Mitigating site effects in covariance for machine learning in neuroimaging data. Human Brain Mapping, 43, 1179–1195. 10.1002/hbm.25688 34904312 PMC8837590

[hbm26708-bib-0016] Chen, A. A. , Luo, C. , Chen, Y. , Shinohara, R. T. , & Shou, H. (2022). Privacy‐preserving harmonization via distributed ComBat. NeuroImage, 248, 118822. 10.1016/j.neuroimage.2021.118822 34958950 PMC9802006

[hbm26708-bib-0017] Crombé, A. , Kind, M. , Fadli, D. , Le Loarer, F. , Italiano, A. , Buy, X. , & Saut, O. (2020). Intensity harmonization techniques influence radiomics features and radiomics‐based predictions in sarcoma patients. Scientific Reports, 10, 15496. 10.1038/s41598-020-72535-0 32968131 PMC7511974

[hbm26708-bib-0018] Das, S. R. , Avants, B. B. , Grossman, M. , & Gee, J. C. (2009). Registration based cortical thickness measurement. NeuroImage, 45, 867–879. 10.1016/j.neuroimage.2008.12.016 19150502 PMC2836782

[hbm26708-bib-0019] Dewey, B. E. , Zhao, C. , Reinhold, J. C. , Carass, A. , Fitzgerald, K. C. , Sotirchos, E. S. , Saidha, S. , Oh, J. , Pham, D. L. , Calabresi, P. A. , van Zijl, P. C. M. , & Prince, J. L. (2019). DeepHarmony: A deep learning approach to contrast harmonization across scanner changes. Magnetic Resonance Imaging, 64, 160–170. 10.1016/j.mri.2019.05.041 31301354 PMC6874910

[hbm26708-bib-0020] Di Martino, A. , Yan, C.‐G. , Li, Q. , Denio, E. , Castellanos, F. X. , Alaerts, K. , Anderson, J. S. , Assaf, M. , Bookheimer, S. Y. , Dapretto, M. , Deen, B. , Delmonte, S. , Dinstein, I. , Ertl‐Wagner, B. , Fair, D. A. , Gallagher, L. , Kennedy, D. P. , Keown, C. L. , Keysers, C. , … Milham, M. P. (2014). The autism brain imaging data exchange: Towards a large‐scale evaluation of the intrinsic brain architecture in autism. Molecular Psychiatry, 19, 659–667. 10.1038/mp.2013.78 23774715 PMC4162310

[hbm26708-bib-0021] Fatania, K. , Clark, A. , Frood, R. , Scarsbrook, A. , Al‐Qaisieh, B. , Currie, S. , & Nix, M. (2022). Harmonisation of scanner‐dependent contrast variations in magnetic resonance imaging for radiation oncology, using style‐blind auto‐encoders. Physics and Imaging in Radiation Oncology, 22, 115–122. 10.1016/j.phro.2022.05.005 35619643 PMC9127401

[hbm26708-bib-0022] Fortin, J.‐P. , Cullen, N. , Sheline, Y. I. , Taylor, W. D. , Aselcioglu, I. , Cook, P. A. , Adams, P. , Cooper, C. , Fava, M. , McGrath, P. J. , McInnis, M. , Phillips, M. L. , Trivedi, M. H. , Weissman, M. M. , & Shinohara, R. T. (2018). Harmonization of cortical thickness measurements across scanners and sites. NeuroImage, 167, 104–120. 10.1016/j.neuroimage.2017.11.024 29155184 PMC5845848

[hbm26708-bib-0023] Fortin, J.‐P. , Parker, D. , Tunç, B. , Watanabe, T. , Elliott, M. A. , Ruparel, K. , Roalf, D. R. , Satterthwaite, T. D. , Gur, R. C. , Gur, R. E. , Schultz, R. T. , Verma, R. , & Shinohara, R. T. (2017). Harmonization of multi‐site diffusion tensor imaging data. NeuroImage, 161, 149–170. 10.1016/j.neuroimage.2017.08.047 28826946 PMC5736019

[hbm26708-bib-0024] Fu, H. , Li, C. , Liu, X. , Gao, J. , Celikyilmaz, A. , & Carin, L. (2019). Cyclical annealing schedule: A simple approach to mitigating KL vanishing.

[hbm26708-bib-0025] Han, X. , Jovicich, J. , Salat, D. , van der Kouwe, A. , Quinn, B. , Czanner, S. , Busa, E. , Pacheco, J. , Albert, M. , Killiany, R. , Maguire, P. , Rosas, D. , Makris, N. , Dale, A. , Dickerson, B. , & Fischl, B. (2006). Reliability of MRI‐derived measurements of human cerebral cortical thickness: The effects of field strength, scanner upgrade and manufacturer. NeuroImage, 32, 180–194. 10.1016/j.neuroimage.2006.02.051 16651008

[hbm26708-bib-0026] Horng, H. , Singh, A. , Yousefi, B. , Cohen, E. A. , Haghighi, B. , Katz, S. , Noël, P. B. , Shinohara, R. T. , & Kontos, D. (2022). Generalized ComBat harmonization methods for radiomic features with multi‐modal distributions and multiple batch effects. Scientific Reports, 12, 4493. 10.1038/s41598-022-08412-9 35296726 PMC8927332

[hbm26708-bib-0027] Hu, F. , Chen, A. A. , Horng, H. , Bashyam, V. , Davatzikos, C. , Alexander‐Bloch, A. , Li, M. , Shou, H. , Satterthwaite, T. D. , Yu, M. , & Shinohara, R. T. (2023). Image harmonization: A review of statistical and deep learning methods for removing batch effects and evaluation metrics for effective harmonization. NeuroImage, 274, 120125. 10.1016/j.neuroimage.2023.120125 37084926 PMC10257347

[hbm26708-bib-0028] Huang, X. , & Belongie, S. (2017). Arbitrary style transfer in real‐time with adaptive instance normalization. 10.48550/arXiv.1703.06868

[hbm26708-bib-0029] Jack, C. R., Jr ., Bernstein, M. A. , Borowski, B. J. , Gunter, J. L. , Fox, N. C. , Thompson, P. M. , Schuff, N. , Krueger, G. , Killiany, R. J. , DeCarli, C. S. , Dale, A. M. , Carmichael, O. W. , Tosun, D. , Weiner, M. W. , & Alzheimer's Disease Neuroimaging Initiative . (2010). Update on the magnetic resonance imaging core of the Alzheimer's Disease Neuroimaging Initiative. Alzheimer's & Dementia, 6, 212–220. 10.1016/j.jalz.2010.03.004 PMC2886577 20451869

[hbm26708-bib-0030] Johnson, W. E. , Li, C. , & Rabinovic, A. (2007). Adjusting batch effects in microarray expression data using empirical Bayes methods. Biostatistics, 8, 118–127. 10.1093/biostatistics/kxj037 16632515

[hbm26708-bib-0031] Jovicich, J. , Czanner, S. , Greve, D. , Haley, E. , van der Kouwe, A. , Gollub, R. , Kennedy, D. , Schmitt, F. , Brown, G. , Macfall, J. , Fischl, B. , & Dale, A. (2006). Reliability in multi‐site structural MRI studies: Effects of gradient non‐linearity correction on phantom and human data. NeuroImage, 30, 436–443. 10.1016/j.neuroimage.2005.09.046 16300968

[hbm26708-bib-0032] Kingma, D. P. , & Ba, J. (2017). Adam: A method for stochastic optimization. 10.48550/arXiv.1412.6980

[hbm26708-bib-0033] Kingma, D. P. , & Welling, M. (2014). Auto‐encoding variational Bayes.

[hbm26708-bib-0034] Klein, A. , & Tourville, J. (2012). 101 labeled brain images and a consistent human cortical labeling protocol. Frontiers in Neuroscience, 6, 171. 10.3389/fnins.2012.00171 23227001 PMC3514540

[hbm26708-bib-0035] Koutsouleris, N. , Davatzikos, C. , Borgwardt, S. , Gaser, C. , Bottlender, R. , Frodl, T. , Falkai, P. , Riecher‐Rössler, A. , Möller, H.‐J. , Reiser, M. , Pantelis, C. , & Meisenzahl, E. (2014). Accelerated brain aging in schizophrenia and beyond: A neuroanatomical marker of psychiatric disorders. Schizophrenia Bulletin, 40, 1140–1153. 10.1093/schbul/sbt142 24126515 PMC4133663

[hbm26708-bib-0036] Lopez, R. , Regier, J. , Cole, M. B. , Jordan, M. I. , & Yosef, N. (2018). Deep generative modeling for single‐cell transcriptomics. Nature Methods, 15, 1053–1058. 10.1038/s41592-018-0229-2 30504886 PMC6289068

[hbm26708-bib-0037] Loshchilov, I. , & Hutter, F. (2019). Decoupled weight decay regularization. 10.48550/arXiv.1711.05101

[hbm26708-bib-0038] Lotfollahi, M. , Wolf, F. A. , & Theis, F. J. (2019). scGen predicts single‐cell perturbation responses. Nature Methods, 16, 715–721. 10.1038/s41592-019-0494-8 31363220

[hbm26708-bib-0039] Maikusa, N. , Zhu, Y. , Uematsu, A. , Yamashita, A. , Saotome, K. , Okada, N. , Kasai, K. , Okanoya, K. , Yamashita, O. , Tanaka, S. C. , & Koike, S. (2021). Comparison of traveling‐subject and ComBat harmonization methods for assessing structural brain characteristics. Human Brain Mapping, 42, 5278–5287. 10.1002/hbm.25615 34402132 PMC8519865

[hbm26708-bib-0040] Manjón, J. V. , Coupé, P. , Martí‐Bonmatí, L. , Collins, D. L. , & Robles, M. (2010). Adaptive non‐local means denoising of MR images with spatially varying noise levels. Journal of Magnetic Resonance Imaging, 31, 192–203. 10.1002/jmri.22003 20027588

[hbm26708-bib-0041] Marek, S. , Tervo‐Clemmens, B. , Calabro, F. J. , Montez, D. F. , Kay, B. P. , Hatoum, A. S. , Donohue, M. R. , Foran, W. , Miller, R. L. , Hendrickson, T. J. , Malone, S. M. , Kandala, S. , Feczko, E. , Miranda‐Dominguez, O. , Graham, A. M. , Earl, E. A. , Perrone, A. J. , Cordova, M. , Doyle, O. , … Dosenbach, N. U. F. (2022). Reproducible brain‐wide association studies require thousands of individuals. Nature, 1–7, 654–660. 10.1038/s41586-022-04492-9 PMC8991999 35296861

[hbm26708-bib-0042] Marek, S. , Tervo‐Clemmens, B. , Nielsen, A. N. , Wheelock, M. D. , Miller, R. L. , Laumann, T. O. , Earl, E. , Foran, W. W. , Cordova, M. , Doyle, O. , Perrone, A. , Miranda‐Dominguez, O. , Feczko, E. , Sturgeon, D. , Graham, A. , Hermosillo, R. , Snider, K. , Galassi, A. , Nagel, B. J. , … Dosenbach, N. U. F. (2019). Identifying reproducible individual differences in childhood functional brain networks: An ABCD study. Developmental Cognitive Neuroscience, 40, 100706. 10.1016/j.dcn.2019.100706 31614255 PMC6927479

[hbm26708-bib-0043] McInnes, L. , Healy, J. , & Melville, J. (2020). UMAP: Uniform manifold approximation and projection for dimension reduction.

[hbm26708-bib-0044] Moyer, D. , Ver Steeg, G. , Tax, C. M. W. , & Thompson, P. M. (2020). Scanner invariant representations for diffusion MRI harmonization. Magnetic Resonance in Medicine, 84, 2174–2189. 10.1002/mrm.28243 32250475 PMC7384065

[hbm26708-bib-0045] Mueller, S. G. , Weiner, M. W. , Thal, L. J. , Petersen, R. C. , Jack, C. R. , Jagust, W. , Trojanowski, J. Q. , Toga, A. W. , & Beckett, L. (2005). Ways toward an early diagnosis in Alzheimer's disease: The Alzheimer's Disease Neuroimaging Initiative (ADNI). Alzheimer's & Dementia, 1, 55–66. 10.1016/j.jalz.2005.06.003 PMC1864941 17476317

[hbm26708-bib-0046] Olson, C. L. (1974). Comparative robustness of six tests in multivariate analysis of variance. Journal of the American Statistical Association, 69, 894–908. 10.1080/01621459.1974.10480224

[hbm26708-bib-0047] Pomponio, R. , Erus, G. , Habes, M. , Doshi, J. , Srinivasan, D. , Mamourian, E. , Bashyam, V. , Nasrallah, I. M. , Satterthwaite, T. D. , Fan, Y. , Launer, L. J. , Masters, C. L. , Maruff, P. , Zhuo, C. , Völzke, H. , Johnson, S. C. , Fripp, J. , Koutsouleris, N. , Wolf, D. H. , … Davatzikos, C. (2020). Harmonization of large MRI datasets for the analysis of brain imaging patterns throughout the lifespan. NeuroImage, 208, 116450. 10.1016/j.neuroimage.2019.116450 31821869 PMC6980790

[hbm26708-bib-0048] Smith, A. , López‐Solà, M. , McMahon, K. , Pedler, A. , & Sterling, M. (2017). Multivariate pattern analysis utilizing structural or functional MRI individuals with musculoskeletal pain and healthy controls: A systematic review. Seminars in Arthritis and Rheumatism, 47, 418–431. 10.1016/j.semarthrit.2017.06.005 28729156

[hbm26708-bib-0049] Sohn, K. , Lee, H. , & Yan, X. (2015). Learning structured output representation using deep conditional generative models. In Advances in neural information processing systems. Curran Associates.

[hbm26708-bib-0050] Takao, H. , Hayashi, N. , & Ohtomo, K. (2011). Effect of scanner in longitudinal studies of brain volume changes. Journal of Magnetic Resonance Imaging, 34, 438–444. 10.1002/jmri.22636 21692137

[hbm26708-bib-0051] Takao, H. , Hayashi, N. , & Ohtomo, K. (2014). Effects of study design in multi‐scanner voxel‐based morphometry studies. NeuroImage, 84, 133–140. 10.1016/j.neuroimage.2013.08.046 23994315

[hbm26708-bib-0052] Trivedi, M. H. , McGrath, P. J. , Fava, M. , Parsey, R. V. , Kurian, B. T. , Phillips, M. L. , Oquendo, M. A. , Bruder, G. , Pizzagalli, D. , Toups, M. , Cooper, C. , Adams, P. , Weyandt, S. , Morris, D. W. , Grannemann, B. D. , Ogden, R. T. , Buckner, R. , McInnis, M. , Kraemer, H. C. , … Weissman, M. M. (2016). Establishing moderators and biosignatures of antidepressant response in clinical care (EMBARC): Rationale and design. Journal of Psychiatric Research, 78, 11–23. 10.1016/j.jpsychires.2016.03.001 27038550 PMC6100771

[hbm26708-bib-0053] Tustison, N. J. , Avants, B. B. , Cook, P. A. , Zheng, Y. , Egan, A. , Yushkevich, P. A. , & Gee, J. C. (2010). N4ITK: Improved N3 bias correction. IEEE Transactions on Medical Imaging, 29, 1310–1320. 10.1109/TMI.2010.2046908 20378467 PMC3071855

[hbm26708-bib-0054] Tustison, N. J. , Holbrook, A. J. , Avants, B. B. , Roberts, J. M. , Cook, P. A. , Reagh, Z. M. , Duda, J. T. , Stone, J. R. , Gillen, D. L. , Yassa, M. A. , & Alzheimer's Disease Neuroimaging Initiative . (2019). Longitudinal mapping of cortical thickness measurements: An Alzheimer's Disease Neuroimaging Initiative‐based evaluation study. Journal of Alzheimer's Disease, 71, 165–183. 10.3233/JAD-190283 PMC10204115 31356207

[hbm26708-bib-0055] Van Essen, D. C. , Smith, S. M. , Barch, D. M. , Behrens, T. E. J. , Yacoub, E. , & Ugurbil, K. (2013). The WU‐Minn Human Connectome Project: An overview. NeuroImage, 80, 62–79. 10.1016/j.neuroimage.2013.05.041 23684880 PMC3724347

[hbm26708-bib-0056] Wager, T. D. , Atlas, L. Y. , Lindquist, M. A. , Roy, M. , Woo, C.‐W. , & Kross, E. (2013). An fMRI‐based neurologic signature of physical pain. New England Journal of Medicine, 368, 1388–1397. 10.1056/NEJMoa1204471 23574118 PMC3691100

[hbm26708-bib-0057] Wang, R. , Chaudhari, P. , & Davatzikos, C. (2021). Harmonization with flow‐based causal inference. Medical image computing and computer‐assisted intervention: MICCAI … International Conference on Medical Image Computing and Computer‐Assisted Intervention 12903, 181–190. 10.1007/978-3-030-87199-4_17 PMC10031749 36961282

[hbm26708-bib-0058] Yu, M. , Linn, K. A. , Cook, P. A. , Phillips, M. L. , McInnis, M. , Fava, M. , Trivedi, M. H. , Weissman, M. M. , Shinohara, R. T. , & Sheline, Y. I. (2018). Statistical harmonization corrects site effects in functional connectivity measurements from multi‐site fMRI data. Human Brain Mapping, 39, 4213–4227. 10.1002/hbm.24241 29962049 PMC6179920

[hbm26708-bib-0059] Zhang, R. , Oliver, L. D. , Voineskos, A. N. , & Park, J. Y. (2022). A structured multivariate approach for removal of latent batch effects. 10.1101/2022.08.01.502396 PMC10503485 37719839

[hbm26708-bib-0060] Zuo, L. , Dewey, B. E. , Liu, Y. , He, Y. , Newsome, S. D. , Mowry, E. M. , Resnick, S. M. , Prince, J. L. , & Carass, A. (2021). Unsupervised MR harmonization by learning disentangled representations using information bottleneck theory. NeuroImage, 243, 118569. 10.1016/j.neuroimage.2021.118569 34506916 PMC10473284

