# Supplementary material for: DeepComBat: A statistically motivated, hyperparameter‐robust, deep learning approach to harmonization of neuroimaging data
Source: Hum Brain Mapp. 2024 Jul 26;45(11):e26708. doi: 10.1002/hbm.26708 (PMC11273293; doi:10.1002/hbm.26708)
Supplement: Supplementary file 1 — DATA S1: Supporting Information. [file HBM-45-e26708-s001.docx]

# Supplemental Materials

Supplemental Table 1. Parametric statistical testing results for two-batch harmonization, reported as negative log 10 p-values. Negative log 10 of conventional p-value threshold 0.05 is 1.30. Larger is more significant. Bolded cells represent the best performance for each column, among harmonization methods that effectively removed batch confounding.

| **Linear regression** – Mean (SD) | | | | | |
| --- | --- | --- | --- | --- | --- |
|  | Batch | Age | Sex | AD Status (CN) | AD Status (LMCI) |
| Raw | 8.70 (7.44) | 15.08 (6.25) | 0.83 (0.76) | 15.51 (7.75) | 6.02 (3.11) |
| ComBat | 0.19 (0.15) | **15.14 (6.27)** | 0.83 (0.76) | 15.55 (7.77) | 6.02 (3.12) |
| CovBat | **0.05 (0.04)** | **15.14 (6.27)** | 0.84 (0.76) | 15.56 (7.80) | 6.05 (3.14) |
| dcVAE | 9.48 (2.63) | 23.68 (3.07) | 0.41 (0.10) | 26.70 (2.89) | 9.27 (1.81) |
| gcVAE | 9.48 (2.86) | 23.46 (3.87) | 0.41 (0.11) | 26.00 (3.69) | 9.01 (1.83) |
| DeepComBat | 0.25 (0.18) | 14.99 (6.31) | **0.85 (0.78)** | **16.16 (8.24)** | **6.18 (3.11)** |
| **Multivariate analysis of variance** | | | | | |
|  | Batch | Age | Sex | AD Status |  |
| Raw | 101.73 | 33.56 | 25.50 | 15.55 |  |
| ComBat | **0** | 31.26 | **22.14** | 15.20 |  |
| CovBat | **0** | 31.27 | 22.01 | 15.35 |  |
| dcVAE | 11.96 | 9.30 | 0.46 | 10.40 |  |
| gcVAE | 10.63 | 13.20 | 0.43 | 11.72 |  |
| DeepComBat | **0** | **32.66** | 18.54 | **17.65** |  |

Supplemental Table 2. Non-parametric statistical testing results for two-batch harmonization. Higher p-value corresponds to less statistically-detectable differences between batches.

|  | **Anderson-Darling** | **kBET** |
| --- | --- | --- |
| Raw | 0.03 (0.13) | 0 |
| ComBat | 0.52 (0.29) | 0 |
| CovBat | **0.56 (0.27)** | 0.166 |
| dcVAE | 0 (0) | 0 |
| gcVAE | 0 (0) | 0 |
| DeepComBat | 0.42 (0.21) | **0.748** |

Supplemental Table 3. Parametric statistical testing results under various DeepComBat hyperparameter values for three-batch harmonization, reported as negative log 10 p-values. Results from training using both cyclic annealing and constant $\lambda$ are provided. Negative log 10 of conventional p-value threshold 0.05 is 1.30. Larger is more significant. Bolded cells represent the best performance for each column, among harmonization methods.

| **Linear regression, Cyclic Annealing** – Mean (SD) | | | | | |
| --- | --- | --- | --- | --- | --- |
|  | Batch | Age | Sex | AD Status (CN) | AD Status (LMCI) |
| $\lambda=0.00625$ | 0.26 (0.21) | 15.02 (6.3) | 0.86 (0.79) | 16.18 (8.20) | 6.19 (3.15) |
| $\lambda=0.025$ | 0.27 (0.18) | 15 (6.32) | 0.84 (0.77) | 16.01 (8.11) | 6.12 (3.06) |
| $\lambda=0.1$ | 0.25 (0.18) | 14.99 (6.31) | 0.85 (0.78) | 16.16 (8.24) | 6.18 (3.11) |
| $\lambda=0.4$ | **0.20 (0.14)** | 15.08 (6.39) | **0.87 (0.81)** | 16.36 (8.30) | 6.21 (3.13) |
| $\lambda=1.6$ | 0.28 (0.18) | **15.23 (6.51)** | **0.87 (0.79)** | **16.46 (8.32)** | **6.29 (3.17)** |
| **Multivariate analysis of variance, Cyclic Annealing** | | | | | |
|  | Batch | Age | Sex | AD Status |  |
| $\lambda=0.00625$ | **0** | 33.38 | 17.77 | 17.40 |  |
| $\lambda=0.025$ | **0** | 32.66 | 18.54 | 17.65 |  |
| $\lambda=0.1$ | **0** | 33.94 | **19.05** | **18.30** |  |
| $\lambda=0.4$ | **0** | **34.35** | 18.72 | 18.17 |  |
| $\lambda=1.6$ | **0** | 34.02 | 18.91 | 17.90 |  |
| **Linear regression, Constant** – Mean (SD) | | | | | |
|  | Batch | Age | Sex | AD Status (CN) | AD Status (LMCI) |
| $\lambda=0.00625$ | 0.21 (0.14) | 15.07 (6.44) | 0.86 (0.81) | 16.28 (8.38) | 6.21 (3.19) |
| $\lambda=0.025$ | **0.07 (0.07)** | 15.03 (6.31) | 0.85 (0.78) | 16.37 (8.27) | 6.41 (3.24) |
| $\lambda=0.1$ | 0.16 (0.11) | 14.85 (6.31) | 0.86 (0.80) | 16.13 (8.30) | 6.28 (3.22) |
| $\lambda=0.4$ | 0.15 (0.11) | 14.86 (6.26) | 0.84 (0.78) | 16.21 (8.15) | 6.28 (3.14) |
| $\lambda=1.6$ | 0.27 (0.13) | **15.38 (6.41)** | **0.88 (0.81)** | **16.88 (8.51)** | **6.56 (3.29)** |
| **Multivariate analysis of variance, Constant** | | | | | |
|  | Batch | Age | Sex | AD Status |  |
| $\lambda=0.00625$ | 0 | 34.98 | 19.06 | 18.07 |  |
| $\lambda=0.025$ | 0 | 33.69 | 18.77 | 17.30 |  |
| $\lambda=0.1$ | 0 | 33.95 | 18.69 | 18.01 |  |
| $\lambda=0.4$ | 0 | 34.42 | 18.29 | 17.15 |  |
| $\lambda=1.6$ | 0 | **34.99** | **20.60** | **18.32** |  |

Supplemental Table 4. Non-parametric statistical testing results under various DeepComBat hyperparameter values for three-batch harmonization. Higher p-value corresponds to less statistically-detectable differences between batches. Bolded cells represent the best performance for each column, among harmonization methods.

| **Cyclic** | **Anderson-Darling** | **kBET** |
| --- | --- | --- |
| $\lambda=0.00625$ | 0.41 (0.23) | 0.623 |
| $\lambda=0.025$ | 0.45 (0.26) | 0.344 |
| $\lambda=0.1$ | 0.42 (0.21) | **0.748** |
| $\lambda=0.4$ | **0.46 (0.22)** | 0.289 |
| $\lambda=1.6$ | 0.44 (0.26) | 0.611 |
| **Constant** | **Anderson-Darling** | **kBET** |
| $\lambda=0.00625$ | 0.36 (0.24) | 0.524 |
| $\lambda=0.025$ | **0.49 (0.24)** | **0.978** |
| $\lambda=0.1$ | 0.37 (0.25) | 0.766 |
| $\lambda=0.4$ | 0.43 (0.25) | 0.891 |
| $\lambda=1.6$ | 0.39 (0.24) | 0.521 |


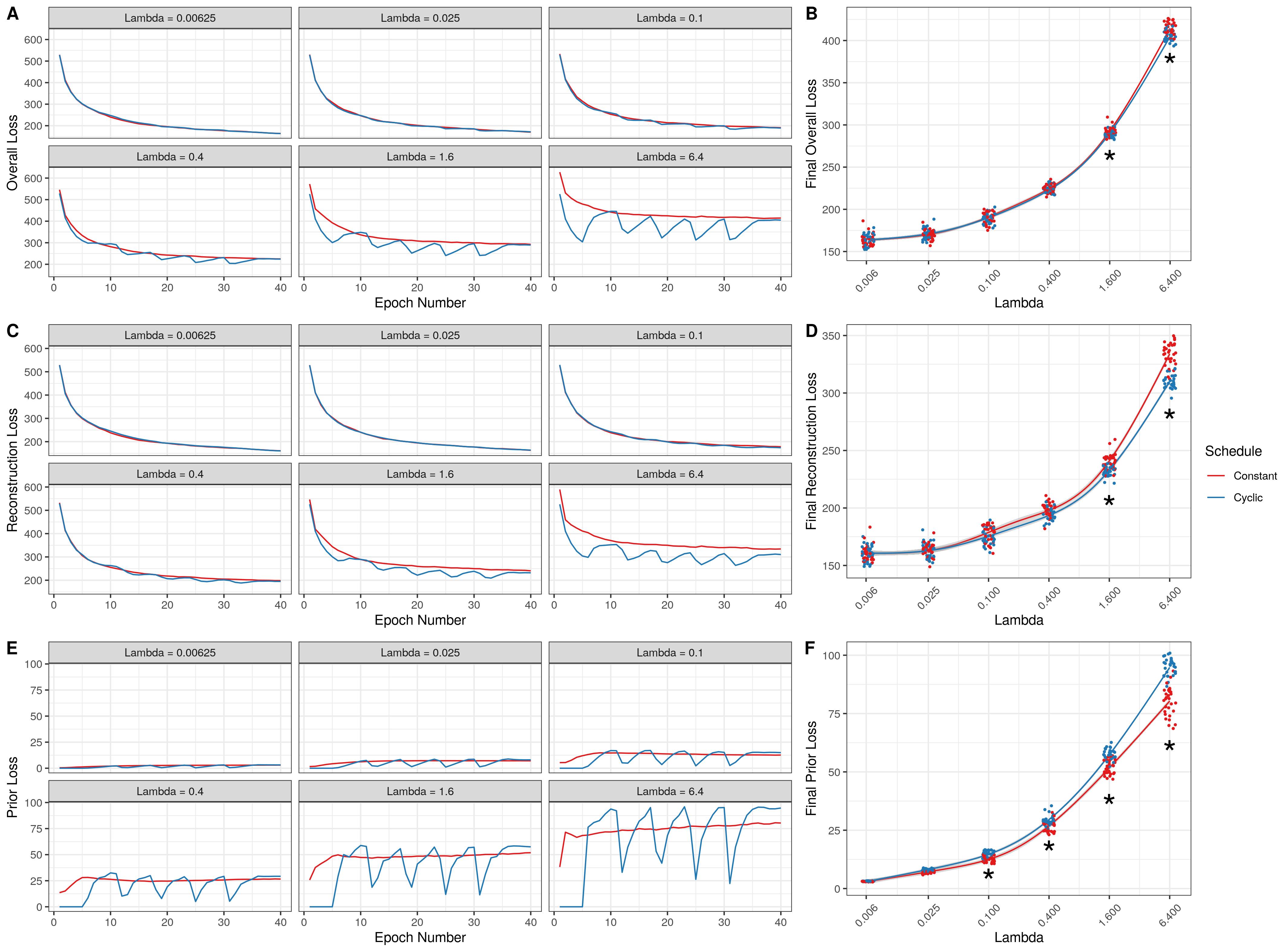


Supplemental Figure 1: Training loss profiles for internal, three-batch harmonization under various DeepComBat hyperparameter values. Red indicates training losses when $\lambda_{\text{Final}}$is held constant throughout training. Blue indicates losses when cyclic annealing is used to vary $\lambda_{\text{Final}}$. Final $\lambda_{\text{Final}}$, and therefore theoretical target, are identical for constant and cyclic annealing. For each pair of $\lambda_{\text{Final}}$ and schedule, 30 independent runs of DeepComBat were performed. In Panels B, D, and F, the x-axis is on a log 2 scale and asterisks indicate significant differences between constant and cyclic annealing schedules at that $\lambda_{\text{Final}}$ (p < 0.05). A: Average overall loss profile across epochs for different $\lambda_{\text{Final}}$. B: Final overall losses per run after the last training epoch for different {}. C: Average reconstruction loss profile across epochs for different $\lambda_{\text{Final}}$. D: Final reconstruction losses per run after the last training epoch for different $\lambda_{\text{Final}}$. E: Average prior loss profile across epochs for different $\lambda_{\text{Final}}$. F: Final prior losses per run after the last training epoch for different $\lambda_{\text{Final}}$.


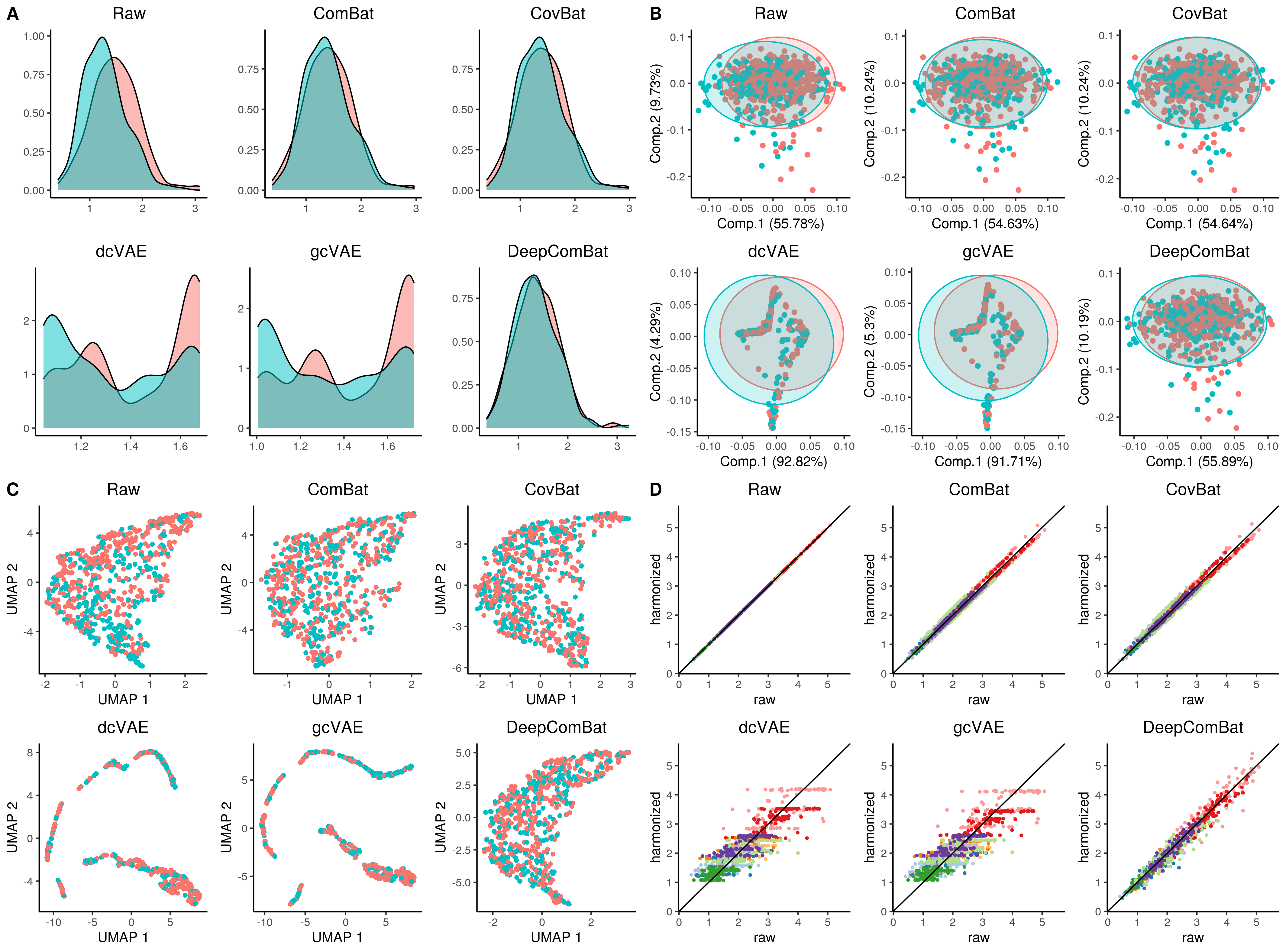


Supplemental Figure 2: Internal harmonization qualitative visualizations for Siemens vs non-Siemens harmonization. In Panels A, B, and C, red corresponds to Siemens and blue corresponds to non-Siemens. A: Density plots of one randomly sampled feature. B: PCA plots, where PCA ellipses denote major and minor axes for each batch, centered at the batch-wise mean. C: UMAP plots. D: Randomly-sampled harmonized values plotted against their corresponding raw values. Colors indicate each of the 10 randomly-sampled cortical thickness features.


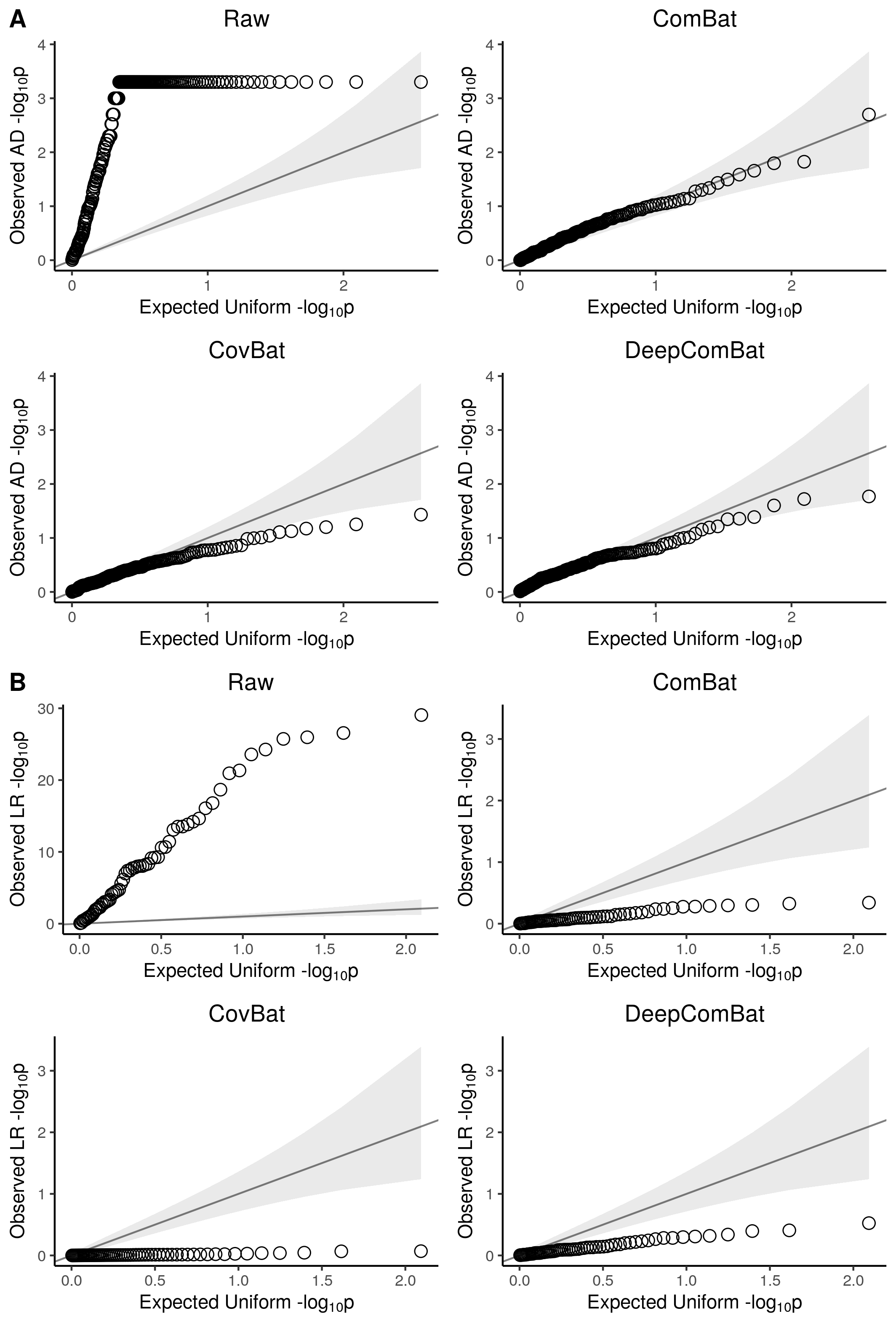


Supplemental Figure 3: Internal harmonization Q-Q plots of observed feature-wise negative log 10 p-values. Observed p-values are plotted against expected negative log 10 p-values under a uniform distribution. Gray band corresponds to 95% confidence intervals for whether observed data was sampled from a uniform. A: Results from Anderson-Darling test. B: Results from linear regression for batch, when other covariates are accounted for. Y-axis scales differ between panels.


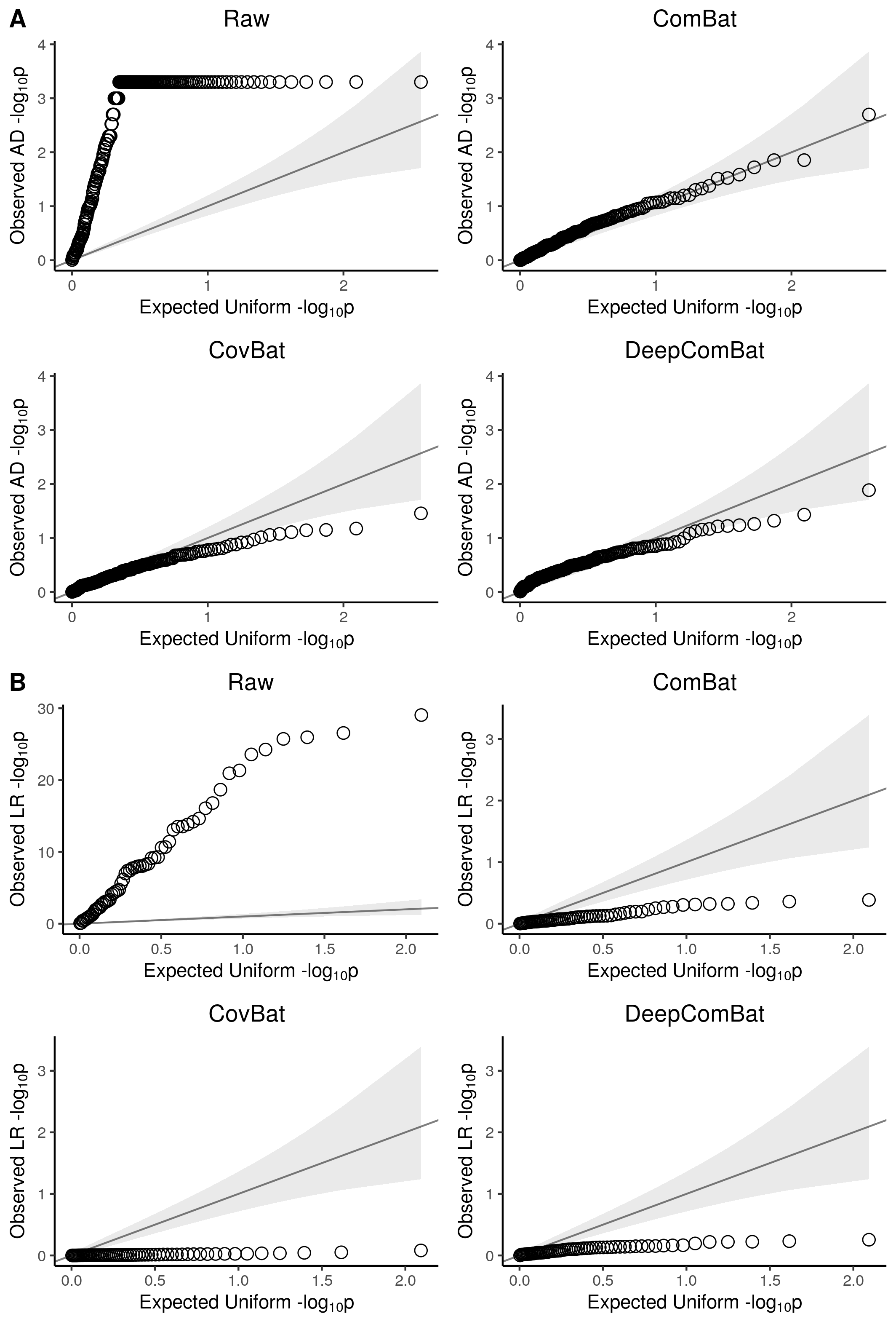


Supplemental Figure 4: External harmonization Q-Q plots of observed feature-wise negative log 10 p-values. Observed p-values are plotted against expected negative log 10 p-values under a uniform distribution. Gray band corresponds to 95% confidence intervals for whether observed data was sampled from a uniform. A: Results from Anderson-Darling test. B: Results from linear regression for batch, when other covariates are accounted for. Y-axis scales differ between panels.


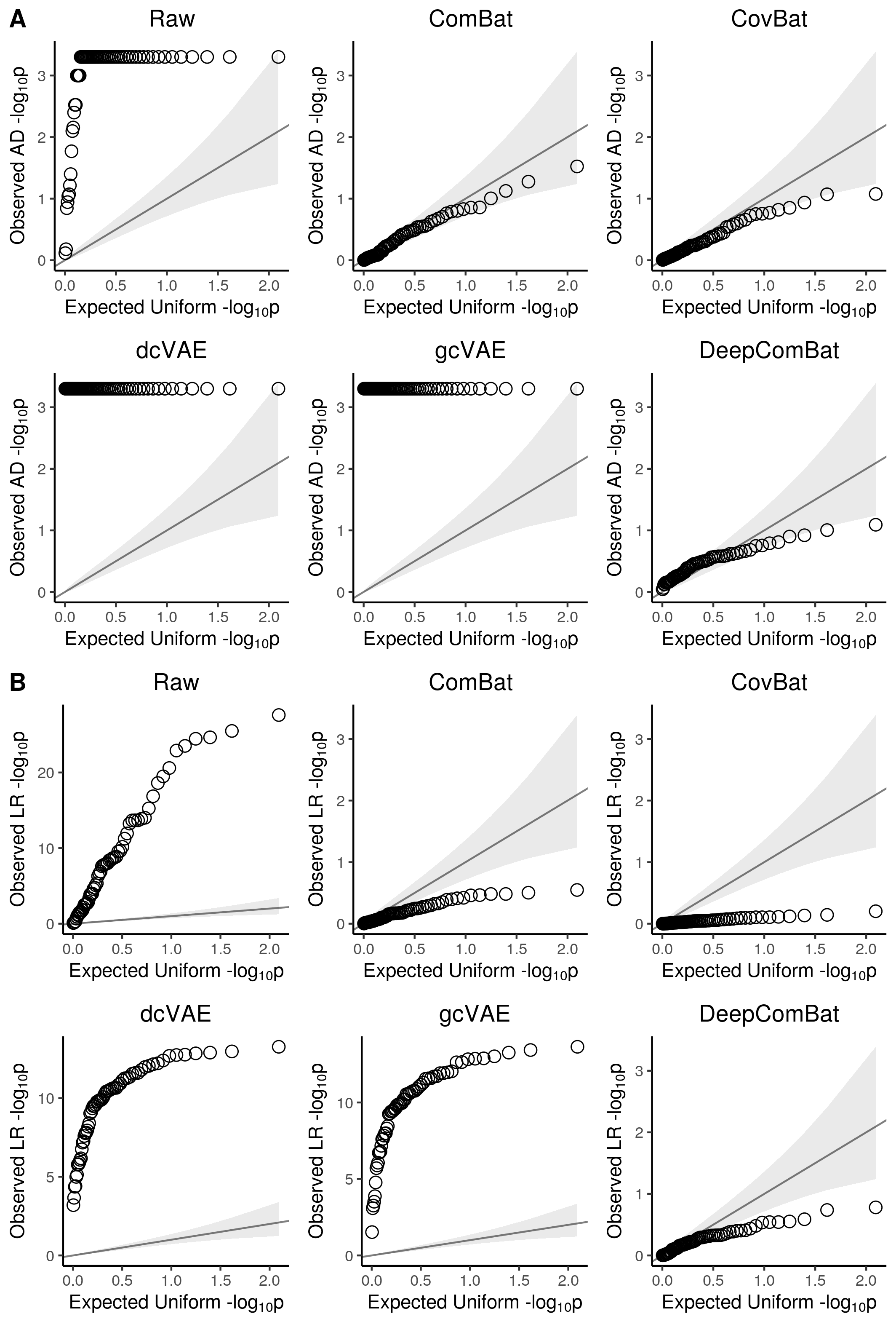


Supplemental Figure 5: Internal harmonization Q-Q plots of observed feature-wise negative log 10 p-values for Siemens vs non-Siemens harmonization. Observed p-values are plotted against expected negative log 10 p-values under a uniform distribution. Gray band corresponds to 95% confidence intervals for whether observed data was sampled from a uniform. A: Results from Anderson-Darling test. B: Results from linear regression for batch, when other covariates are accounted for. Y-axis scales differ between panels.


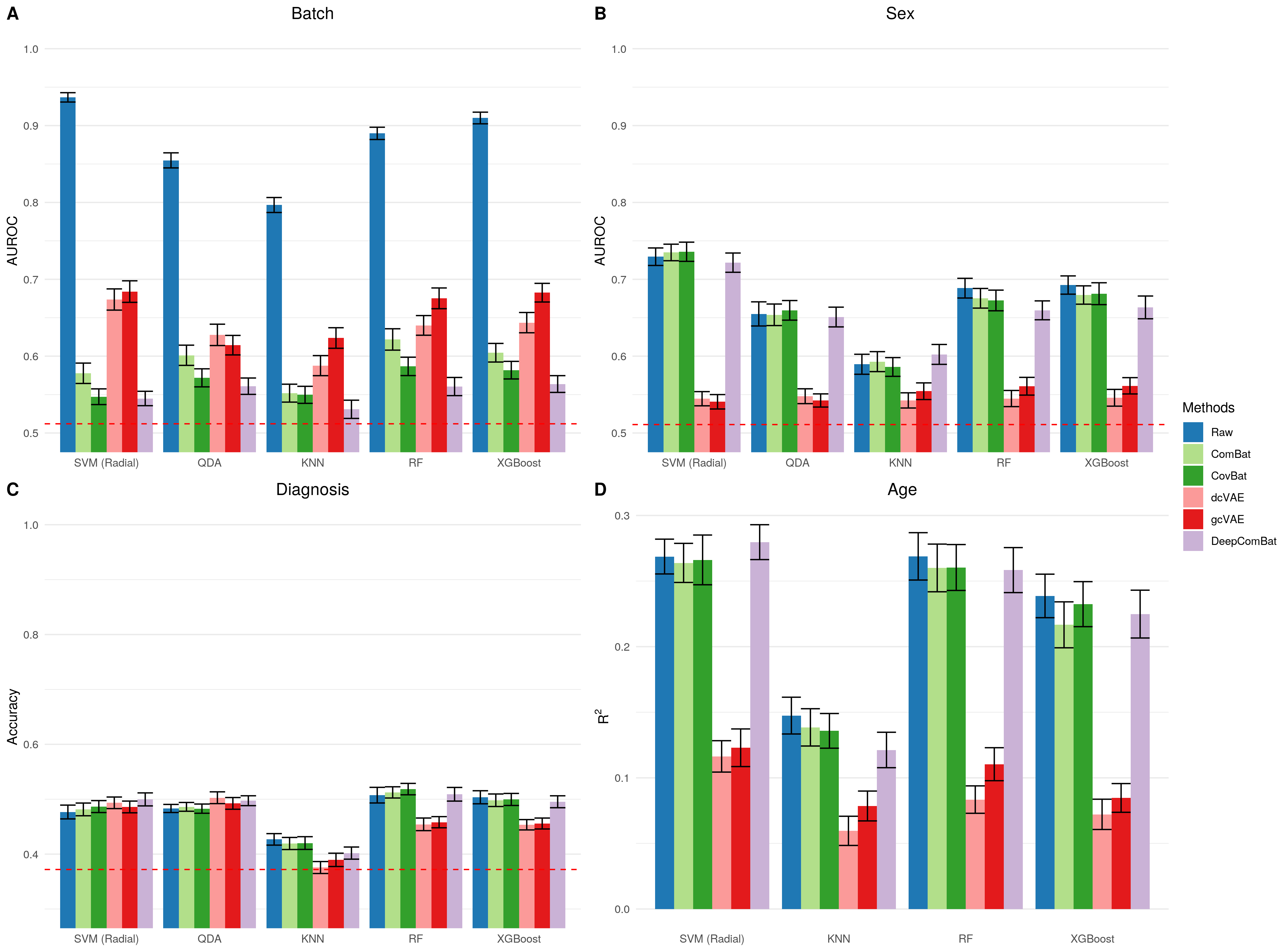


Supplemental Figure 6: Internal harmonization machine learning results for Siemens vs non-Siemens harmonization. Validation set performance metrics are shown for ten repeats of 10-fold cross validation. Error bars correspond to 95% confidence intervals. A: Average AUROC for predicting batch. Lower is better. B: Average AUROC for predicting sex. C: Average accuracy for predicting Alzheimer disease status. D: Average $R^{2}$ value for predicting age.


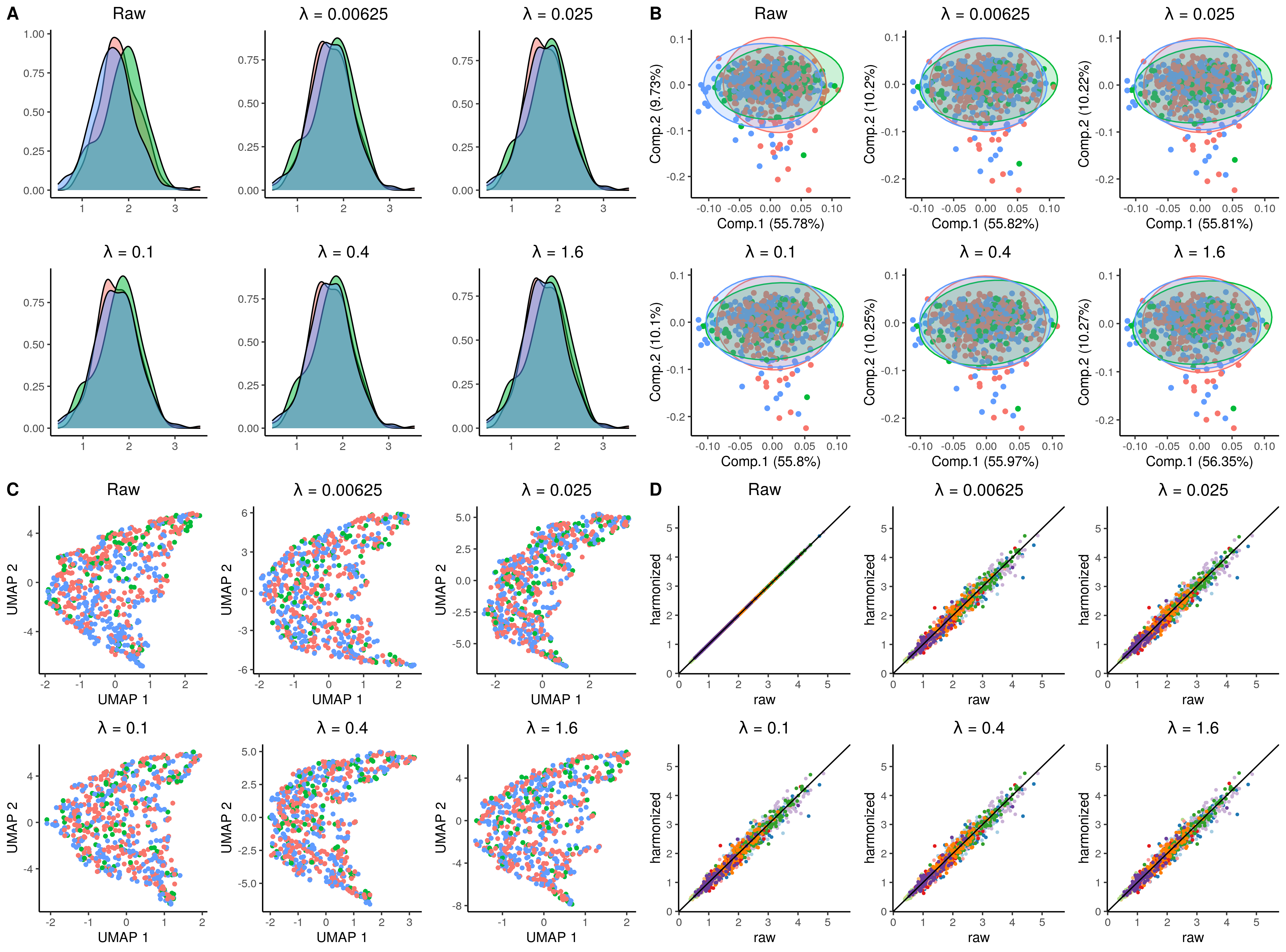


Supplemental Figure 7: Internal harmonization qualitative visualizations under various DeepComBat hyperparameter values for three-batch harmonization. In Panels A, B, and C, red corresponds to Siemens, green corresponds to GE, and blue corresponds to Philips. A: Density plots of one randomly sampled feature. B: PCA plots, where PCA ellipses denote major and minor axes for each batch, centered at the batch-wise mean. C: UMAP plots. D: Randomly-sampled harmonized values plotted against their corresponding raw values. Colors indicate each of the 10 randomly-sampled cortical thickness features.


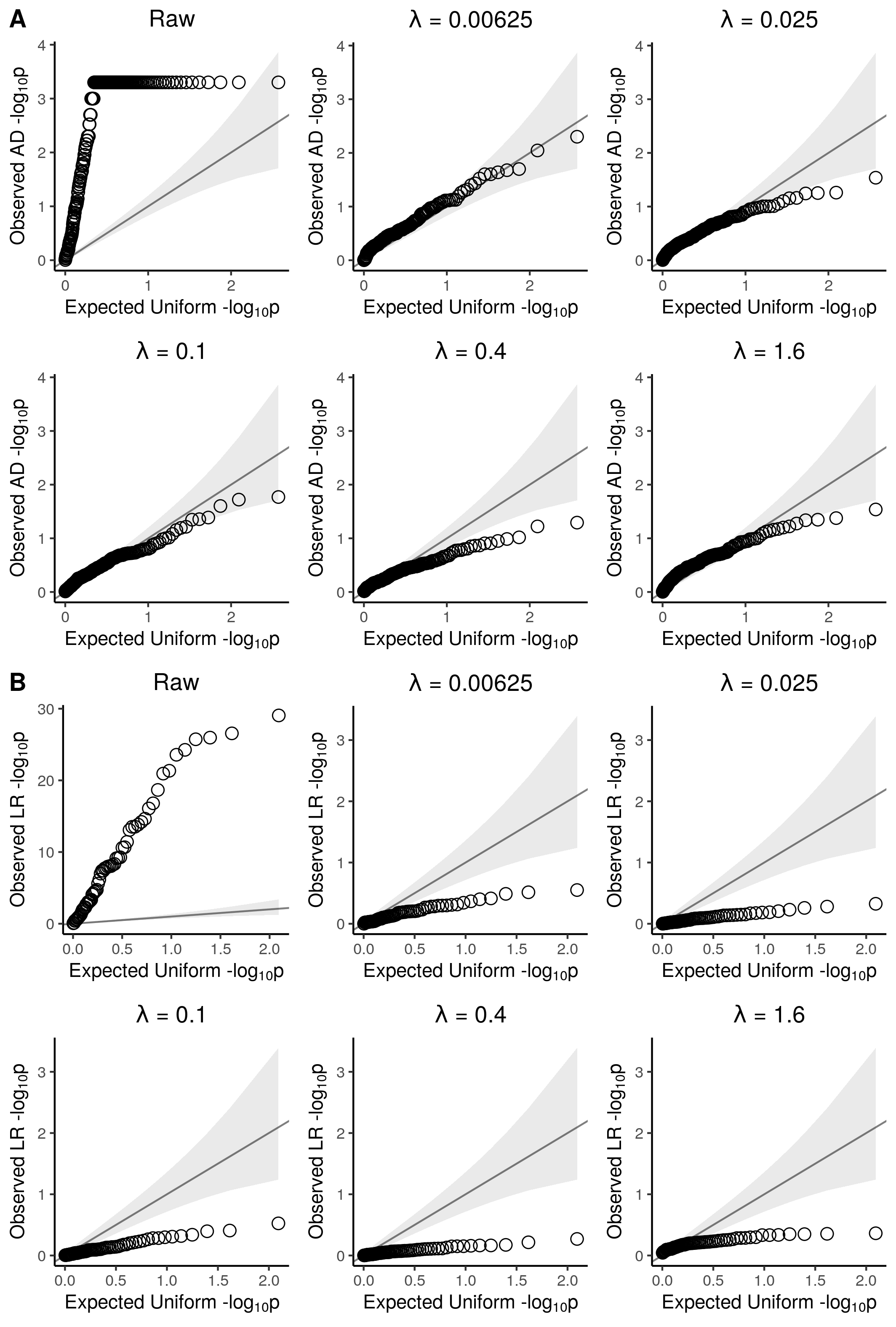


Supplemental Figure 8: Internal harmonization Q-Q plots of observed feature-wise negative log 10 p-values under various DeepComBat hyperparameter values for three-batch harmonization. Observed p-values are plotted against expected negative log 10 p-values under a uniform distribution. Gray band corresponds to 95% confidence intervals for whether observed data was sampled from a uniform. A: Results from Anderson-Darling test. B: Results from linear regression for batch, when other covariates are accounted for. Y-axis scales differ between panels.


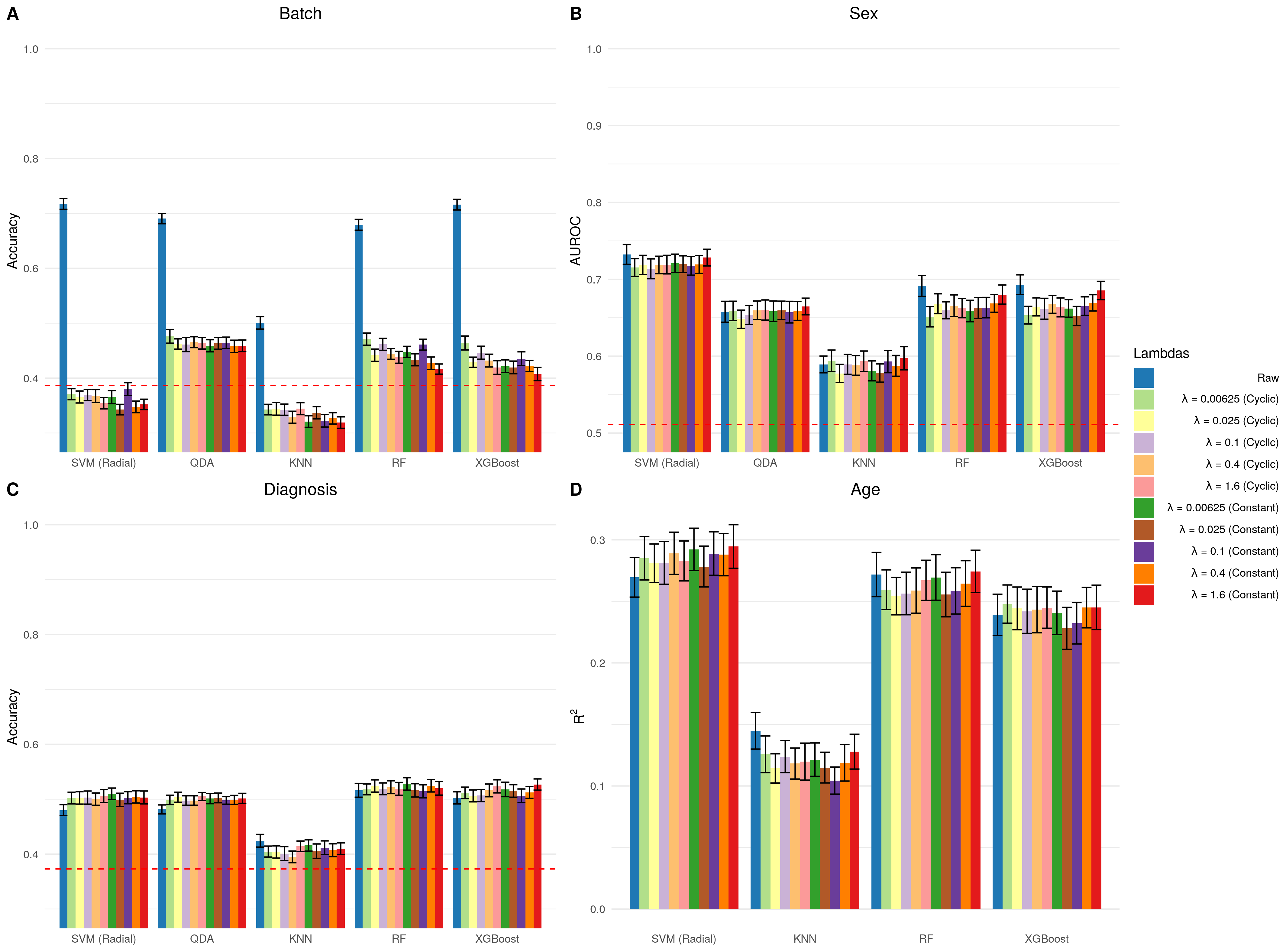


Supplemental Figure 9: Internal harmonization machine learning results under various DeepComBat hyperparameter values for three-batch harmonization. Validation set performance metrics are shown for ten repeats of 10-fold cross validation. Error bars correspond to 95% confidence intervals. Dashed red lines display expected performance of a weighted random classifier. A: Average accuracy for predicting batch. Lower is better. B: Average AUROC for predicting sex. C: Average accuracy for predicting Alzheimer disease status. D: Average $R^{2}$ value for predicting age.
